# Validation of a 3D Camera System for Cycling Analysis

**DOI:** 10.3390/s21134473

**Published:** 2021-06-30

**Authors:** Robson Dias Scoz, Thiago Roberto Espindola, Mateus Freitas Santiago, Paulo Rui de Oliveira, Bruno Mazziotti Oliveira Alves, Luciano Maia Alves Ferreira, César Ferreira Amorim

**Affiliations:** 1 Master and Doctoral Programs in Physical Therapy, Universidade Cidade de Sao Paulo (UNICID), Sao Paulo 03071-000, Brazil; oliveira@netmogi.com.br (P.R.d.O.); brunomazziotti@hotmail.com (B.M.O.A.); cfaemg@gmail.com (C.F.A.); 2 Physical Therapy Department, Universidade do Sul de Santa Catarina (UNISUL), Palhoça 88137-272, Brazil; tii18@hotmail.com (T.R.E.); mateus.freitastkd@gmail.com (M.F.S.); 3 KinesioLab, Instituto Piaget, 8300-025 Silves, Portugal; luciano.maia@silves.ipiaget.pt; 4 Laboratoire de Recherche BioNR, Université du Quebec a Chicoutimi, Saguenay, QC G7H 2B1, Canada; 5 Human Performance Laboratory, Physical Therapy Department, Florida International University—FIU, Miami, FL 33199, USA

**Keywords:** cycling, bicycle, bike fit, bike fitting, kinematic

## Abstract

Background: Kinematic analysis aimed toward scientific investigation or professional purposes is commonly unaffordable and complex to use. Objective: The purpose of this study was to verify concurrent validation between a cycling-specific 3D camera and the gold-standard 3D general camera systems. Methods: Overall, 11 healthy amateur male triathletes were filmed riding their bicycles with Vicon 3D cameras and the Retul 3D cameras for bike fitting analysis simultaneously. All 18 kinematic measurements given by the bike fitting system were compared with the same data given by Vicon cameras through Pearson correlation (*r*), intraclass correlation coefficients (ICC), standard error measurements (SEM), and Bland–Altman (BA) analysis. Confidence intervals of 95% are given. Results: A very high correlation between cameras was found on six of 18 measurements. All other presented a high correlation between cameras (between 0.7 and 0.9). In total, six variables indicate a SEM of less than one degree between systems. Only two variables indicate a SEM higher than two degrees between camera systems. Overall, four measures indicate bias tendency according to BA. Conclusions: The cycling-specific led-emitting 3D camera system tested revealed a high or very high degree of correlation with the gold-standard 3D camera system used in laboratory motion capture. In total, 14 measurements of this equipment could be used in sports medicine clinical practice and even by researchers of cycling studies.

## 1. Introduction

The increasing popularity of cycling as a method of transportation, recreation, and sport has led to an increase in the incidence of musculoskeletal injuries related to its practice [1]. These injuries often occur due to incorrect cyclist posture on the bicycle, as a product of an incorrect equipment configuration or setup with rider’s body measurements and physical conditions [2]. This ergonomic adjustment of bicycle components to the anthropometric measurements of the cyclist, aiming at more comfort, less pain and musculoskeletal overloads from repetitive cycling gestures, is known as “Bike fit”, or “Bike fitting” [3,4].

There are currently different bike fitting methods, but few of them follow a standardized protocol, limiting comparability, and reliability. The majority of bike fitting methods used today rely on anthropometric and kinematic measurements, to analyze static body dimensions and the cyclist dynamic riding posture, respectively. To the best of our knowledge, none use scientific validated measurement instruments to analysis kinematic data, as high-speed 3D cameras are unaffordable to a sport cycling teams, bike fitting services, or bike shops. The need for an accurate measure system in bike fitting relies on injury risk and performance factors, as small changes in posture can produce different power output and aerodynamics [5,6,7].

During the last decade, affordable 3D camera systems aimed to evaluate cycling become more common. One of the most known 3D camera systems used for bike fitting is the Vantage 3D camera system, manufactured by Retul company, which have a standardized training program aimed to its users. Aided by inertial active-maker LED-emitting infrared harness, this portable 3D camera system has become very popular between athletes, mechanics, and bike fitters. As data are displayed in real time, adjustments can be done on the bicycle while changes occur on a rider’s body. An impossible task while using conventional laboratory high speed 3D cameras.

However, this system uses a slow speed infrared camera (18 Hz) generating doubts about its accuracy even when capturing marker’s location on the slow-speed activity of pedaling. Nevertheless, considering the portability factor, real time data response, and the standardization process of use, this 3D camera system could bring advantages to scientific community, cycling practitioners, and professionals, mostly bike fitters.

Therefore, the purpose of this study is to verify concurrent validation of a commercial bike fitting mixed-sensor LED-emitting 3D camera system with the gold-standard laboratorial 3D camera system. Our hypothesis is that a specific 3D camera system could be used as a kinematic tool with a reasonable accuracy when compared to the gold-standard.

## 2. Material and Methods

### 2.1. Design

**Figure 1:**
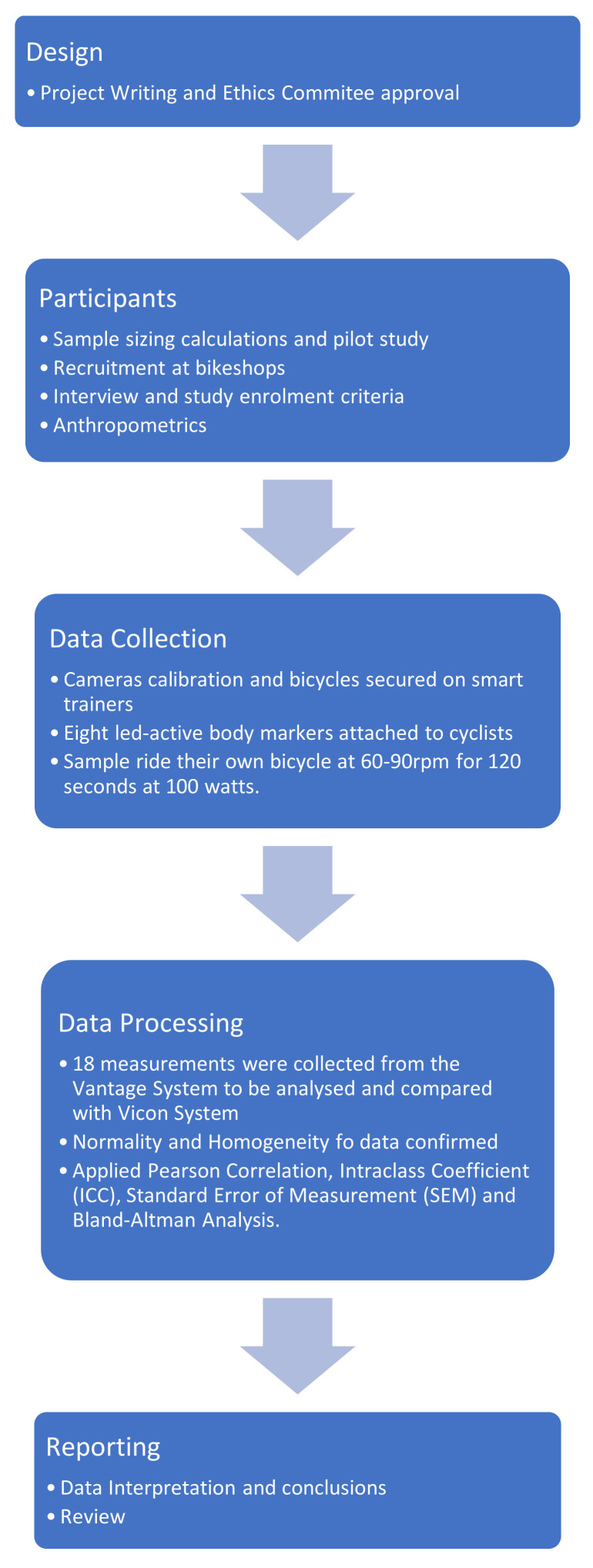
Flowchart of research.

This is a prospective validation preliminary study using data from a 3D camera system borrowed from a professional bike fitter not enrolled in the study. This research report followed the recommendations of the strengthening the reporting of observational studies in epidemiology (STROBE statement) [8] and its design followed the recommendations of the improving healthcare decisions task force (ISPOR database recommendations) [9]. Figure 1 illustrates this study process and stages.

The study was carried out according to the Declaration of Helsinki, following the ethical standards in sports and exercise science research [10]. A protocol was fully approved by the university human research ethics committee of our university with number #39223556. There was no involvement from patients or members of the public in the design, or conduct, or reporting, or dissemination plans of the research.

### 2.2. Participants

Participant sample size followed the examples of 8 to 15 subjects used in similar past researches and validation studies with indoor cycling [11,12,13,14]. A pilot study was conducted with one additional cyclist to previously standardize all procedures. We decided to finish our candidate’s acceptance when we had 11 amateurs, adult male cyclists, in that way reducing statistical damage from possible dropouts. Demographic and anthropometric information of the sample are presented in Table 1.

The participants were recruited through advertisements in local bicycle shops. They were classified as amateurs according to a recent categorization based on weekly training and practice volume in kilometers [15]. They should have more than six months familiarity with their current triathlon bicycle, and answer the physical activity readiness questionnaire (PARQ). Two positive responses to this questionnaire were a criterion for exclusion. Other exclusion criteria were cyclists with post-operative complaints; subjects using pain medication; and candidates younger than 18 years old.

The purpose, experimental procedures, possible risks and benefits of the study were explained to the candidates, who provided a written informed consent form to confirm participation in the study. Participants personal data were deleted after extraction to guarantee anonymity. Final data were stored on a password secure, internet cloud-based website, to avoid risk of information leak or lost. Participants personal data were deleted after extraction to guarantee anonymity. Final data were stored on a password secure, internet cloud-based private website, to avoid risk of information leak or lost.

### 2.3. Instruments

For data collection, three high-speed infrared tridimensional cameras (Bonita Camera System, Vicon Inc., Oxford, UK) were used, capturing infrared-sensitive markers positioned on eight cyclist’s body landmarks. Vicon-Bonita cameras were set to film at 240 Hz and are considered the gold-standard of 3D kinematic analysis in sports biomechanics with an accuracy of 0.4 mm maximal error of measurement. Vicon Bonita Hardware technical specifications can be accessed in the Supplementary Material.

Simultaneously, a mixed inertial-sensor and led-emitting infrared tridimensional camera system (Vantage Camera System, Retul Inc., Boulder, CO, USA) used in bike fitting (also known as Retul 3D Cameras) were used to capture the same anatomical landmarks. According to manufacturer, the system is set to film at 18 Hz with built-in interpolation processes to improve accuracy of the measurements. Complete Retul Vantage Hardware technical specifications can be accessed in the Supplementary Material.

Both cameras captured the same active LED-emitting markers partially covered with infrared-reflexive tape. All system’s calibration followed manufacturer’s manual instructions. Vicon cameras use an active led emitting “T” wand for calibration between subjects, while Vantage system self-calibrates using its combination of inertial sensors and 3D cameras.

Each participant own bicycle was connected to a hydraulic indoor direct-drive smart trainer (Suito, Ellite, Italy), equipped with a built-in power meter. A set of common mechanical tools (like screwdrivers and hex keys) was used to adjust and modify bicycle components.

For data storage and processing, a MacBook Pro Notebook (Cupertino, CA, USA) was used equipped with Microsoft Office software package for Mac (version 2011, Redmond, WA, USA) and statistical package for social sciences (SPSS) from IBM (Armonk, NY, USA). All motion capture raw data were extracted and processed by its own dedicated software’s (Vicon Tracker Software v3.6.1 and Vantage Software v7.0). The variables were imported into a custom-made script in MATLAB^®^ (MathWorks Inc., Natick, MA, USA) for digital filtering of data (second order, zero lag, low pass Butterworth with cut of frequency of 5 Hz) and partition in five consecutive crank cycles. Because the natural frequency of cycling movement was approximately 1 Hz (i.e., for 60 rotations per minute or rpm), we have chosen 5 Hz for kinematics filter in agreement with the minimum sampling frequency of 2.4 times the event frequency, as per the Nyquist theorem commonly used in kinematic studies of cycling [16].

### 2.4. Procedure

**Figure 2:**
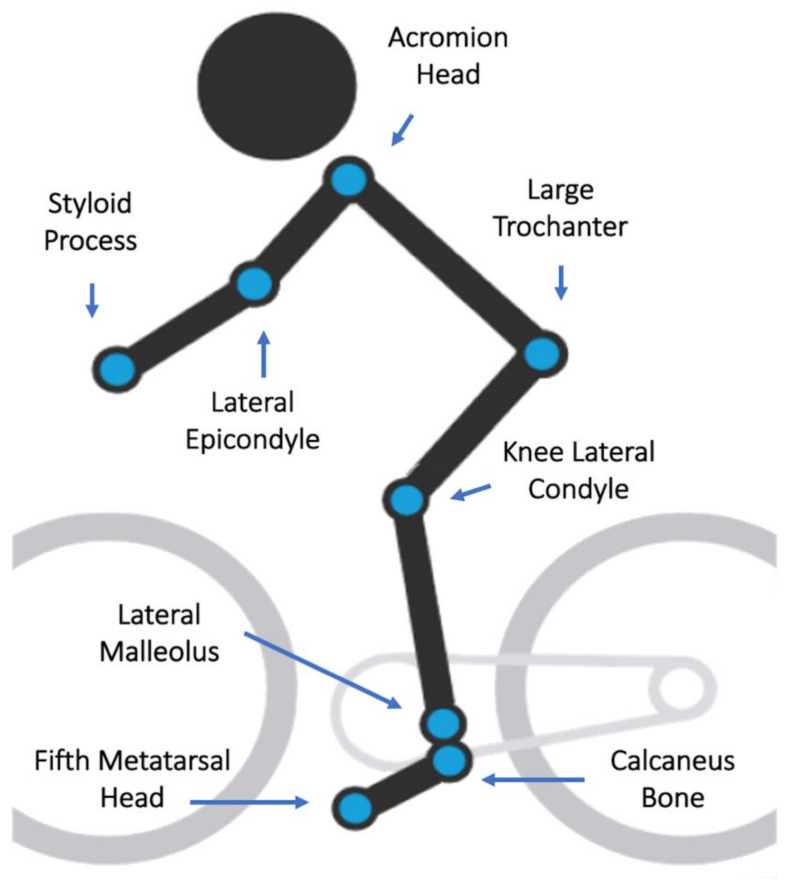
Body markers for 3D kinematic tracking.

Participants were requested to bring their bicycle to the clinic on a convenient pre-determined schedule between 8 am and 12 am from Monday to Friday. They receive a list of recommendations including wearing proper cycling clothing and shoes; do not practice strenuous exercise up to 6 h before a bike fit session; and avoid fasting 3 h before a session. During a session, they are allowed to drink fresh water on demand. The indoor temperature was maintained in 23 degrees Celsius, and humidity levels between 68% and 80%. The same physiotherapist, with 7 years of experience, performed all analysis. Both 3D camera systems filmed the rider’s right side using the same body landmarks. Figure 2 shows the landmarks used to attach each marker on cyclist’s body. Vantage camera systems do not use cluster-markers.

Upon arrival, participants were provided with appropriate explanation and demonstration of all procedures. Cyclists informed their personal data, level of experience with the current bicycle, weekly mileage, objectives, expectations, and complaints. Anthropometric data were recorded before a session began, following International Society for the Advancement of Kineanthropometry (ISAK) Level 1 certified anthropometrist protocol [17]. The participants were then subjected to a standardized motion capture session while pedaling their own bicycles on the smart trainer. The motion capture session had a duration of 120 s. 

After interview and physical assessment, began a standardized motion capture protocol. Each participant’s bicycle was positioned on the trainer while the rider’s body was marked with a harness containing active and passive markers for kinematic tracking. The subjects were asked to ride their bicycles on trainer for 120 s, at 60–90 rpm, with an automatic controlled load of 100 watts [18]. They could then, drop from the bike and rest, while all data from Vicon and Retul cameras were safely stored.

We used Retul measurement descriptions (Table 2) to reproduce these on Vicon software. Retul measurements use 5 references to calculate their measurements. Horizontal level and four crank positions: top, front, down, and rear, respectively, in degrees as zero, 30, 60, and 90. As both cameras filmed the cyclist at the same time, the time frame was synchronized, and we could identify the exact instant of each position on Vicon graphs time per position.

After calibration and identification of all 8 markers, the 18 measurements were triangulated, tracked during the pedaling session and extracted. Then we used the mean angular values between the time-frame window of 60 to 70 s to select Vicon measurements while Retul measurements are given by its own software in real time from the same 10 s time frame. Raw data from Vicon cameras were exported from Vicon software to an Excel spreadsheet. Then we selected the chosen time frame to analyze the mean values.

The active LED-emitting marker of Retul system is round and with a similar size to passive markers used in Vicon motion capture. The center of the Retul marker has a small emitting LED (0.2 mm in diameter), so we applied reflective tape around it to film both systems at the same time. As Vicon uses the center of the maker to calculate its measurements, it would have a clinically unimportant offset, if any, when compared to Retul measurements.

**Figure 3:**
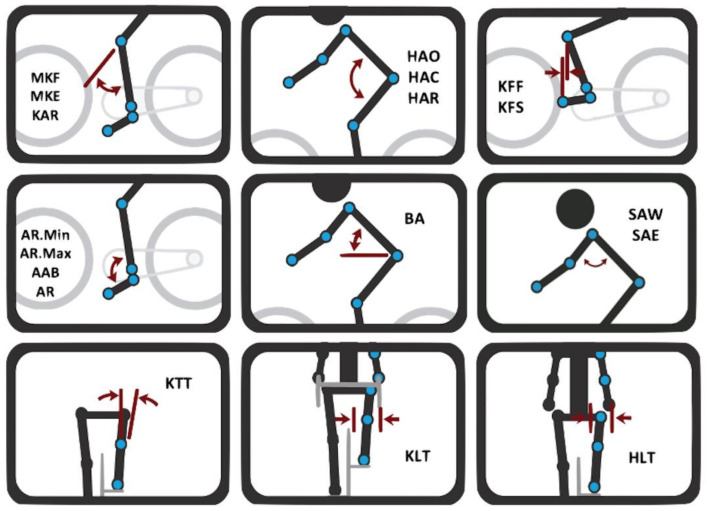
Kinematic measurements.

During motion capture, 18 kinematic measurements were collected. Table 2 shows all 18 measurements’ descriptions, their names, abbreviations and commonly angular ranges. Figure 3 shows a schematic layout of all measurements with rider body markers. Although more measurements are given by Retul-Vantage system, we decided to choose the most used measures in clinical practice by bike fitters and sports medicine professionals.

### 2.5. Data Analysis

Demographic data extracted were: sex, age, height, weight, wingspan, BMI, experience (familiarity) with the current bicycle in months, rider training (practice) in kilometers per month. These data were recorded for descriptive analysis (mean ± standard deviation (SD)). Table 1 shows all demographic and anthropometric data of the sample.

Normality of all data was confirmed using visual inspection and the Kolmogorov–Smirnov test. Homogeneity of variance was assessed via Levene’s Test. Pearson correlation (abbreviated as “*r*”), Intraclass Correlation Coefficient (ICC) were used to identify statistically significant differences between all 18 kinematic measure used.

Bland–Altman analysis was used to identify possible bias tendency in each measure between systems. The student *t*-test for the measure difference between systems (showed as “*p*” on results table) and the linear regression analysis (abbreviated as “*p*” and “reg”, respectively) were calculated and its scatter plots are provided as Supplementary Material.

Confidence intervals (95%) are provided for all measurements. The degree of correlation was classified as very high (higher than 0.9), high (between 0.7 and 0.9). moderate (between 0.5 and 0.7), low (between 0.3 and 0.5), and very low (between 0 and 0.3) [19]. Standard error measurement (SEM) was calculated using a customized Microsoft Excel spreadsheet. All data were processed using a SPSS v.20 (IBM, Chicago, IL, USA) with a level of statistical significance set at alpha level *p* < 0.05.

## 3. Results

Descriptive and inferential analysis results are presented on Table 3. Bland–Altman results are also provided in Table 3, and its 18 scatter plots can be accessed as Supplementary Material.

The “*p*” value on Table 3 is the statistical result of a student *t*-test between means of both systems kinematic measurements. The student *t*-test results demonstrate that all 18 kinematic variables have no statistical difference between both camera systems (*p* < 0.05).

A very high correlation between cameras was found on these measurements: Hip Angle Open (0.91), Shoulder Angle to Wrist (0.90), Shoulder Angle to Elbow (0.91), Knee Forward of Foot (0.94), Knee Forward of Spindle (0.95), Hip Lateral Travel (0.91). All other measurements presented a high correlation between cameras (between 0.7 and 0.9).

The six variables indicate a standard error of measurement (SEM) of less than one degree between both camera systems. They are: Ankle Minimum (0.8), Ankle Range (0.9), Maximum Knee Flexion (0.3), Maximum Knee Extension (0.5), Hip Angle Range (0.5), Knee Travel Tilt (0.3). Only two variables indicate a SEM higher than two degrees between camera systems. They are: Knee Lateral Travel (2.1) and Shoulder Angle to Wrist (2.7). All other 10 variables indicate a SEM between one degree and two degrees.

The Bland–Altman analysis indicated 4 measures with bias tendency when calculated their linear regression: Ankle Range (AR), Knee Angle Range (KAR), Knee Lateral Travel (KLT), and Hip Lateral Travel (HLT). All other 14 measures showed no tendency of bias.

## 4. Discussion

The purpose of this study was to verify concurrent validation of a commercial bike fitting tridimensional led-emitting low-speed 3D camera system with a gold-standard high-speed 3D camera system. Our hypothesis was that an affordable, portable 3D camera system could be used as a kinematic tool with a reasonable accuracy when compared to the gold-standard.

As our results have revealed, all kinematic measurements have a high or very high degree of correlation between Retul/Vantage and Vicon/Bonita cameras. These results indicate a reliable use of the system for indoor cycling kinematic analysis, like commercial bike fitting or even scientific investigations, considering the lower cost, potability and simplicity of the dedicated camera software and hardware. Other researchers have compared bike fitting methods before, but few of them used the gold-standard 3D camera systems [20,21].

Retul/Vantage 3D cameras do not have the same high-speed frame rate of Vicon 3D cameras, as the difference in prices are evident. Even so, this possible difference in frequency acquisition did not impact the precision of this portable system when compared with gold-standard cameras. This could be a result of mathematical interpolations using the crank arm length as a circumference radius. Different from a gait or running analysis, cycling has few secondary body movements beyond the primary motor-effort of lower limbs at sagittal plane. Considering a cadence of 60 rotations per minute, an acquisition frequency of 60 Hz should be able to capture 60 dots along all crank arm circumference [22,23,24]. Using 18 Hz and interpolations from a couple of revolutions, this could generate sufficient information needed to calculate all 18 kinematic measurements and display it in real time for the user. Our study used a cadence between 70 and 90 rpm, where the majority of cycling training regimes occur, so it is unclear if higher cadences can be used with this system [25,26]. As Bland–Altman analysis have demonstrated, four measures presented tendency of bias to upper or lower limits of standard deviations; and should be used with cautious by sports professionals, like bike fitters. Two of these measures are means of range of motions, calculated from another two measures of upper and lower range of motion limits. Their tendency of bias may be related to this calculation as a secondary measure and not a directly extracted from filming measure.

To the best of our knowledge, only three scientific papers have tried to validate a similar camera system. The most similar to our study design and methods had only three subjects and their data were limited to 15 measurements that do not reconstruct all cycling movements, or the relationship between rider and bicycle. Similar to our results, they found high levels of correlation between a LED-emitting camera system for cycling analysis and the gold standard high-speed Vicon cameras [27].

In another research, the authors established the validity and reliability of three different kinematic methods for bike fitting: Vicon cameras as comparison gold standard, one high-speed 2D camera and one electrogoniometer [21]. Images from the 2D camera were separated in frame blocks and analyzed through a generic motion analysis software. They found high correlation between both cameras but the electrogoniometer did not show similar measurements. Although a 2D camera offer a cost-effective alternative to analyze a cyclist kinematically, the post-filming process of digital analysis on a computer frame by frame is too much impeditive and laborious to most bike fitters and researchers.

## 5. Conclusions

This affordable and portable mixed inertial-sensor, led-emitting tridimensional camera system revealed a high and very high degree of correlation with the gold-standard indoor laboratorial tridimensional camera system. Standard error of measurements of less than two degrees was found in 16 of 18 kinematic measurements.

The measurements presented by this system have an acceptable level of accuracy for both bike fitting analysis and cycling research. With a dedicated software, real time motion capture analysis and up to 18 full body common cycling measurements, this system could improve the speed of studies about cycling biomechanics. Reducing long periods of data management.

## 6. Limitations

Our study used a free cadence between 70 and 90 rotations per minute, a common cadence used in cycling training. It is unknown if higher cadences would impact the accuracy of this 3D camera system.

## Figures and Tables

**Table 1:** Demographic and anthropometric characteristics of the sample (values with means ± standard deviations).

Age (years)	38.71	±8.00
Height (m)	1.74	±7.83
Wingspan (m)	1.75	±8.05
Body Mass (kg)	77.62	±10.82
BMI (kg/m^2^)	25.64	±1.78
**Rider Familiarity with Current Bicycle**		
7 to 24 Months	*n* = 7 (64%)	
More than 24 Months	*n* = 4 (36%)	
**Rider Training Volume**		
200 to 400 km/month	*n* = 9 (80%)	
400 to 800 km/month	*n* = 2 (20%)	

**Table 2:** Measurements and joint angular ranges for cycling 3D kinematics analysis.

Measurement	Abbreviation	Angular Range	Description
Ankle Minimum	AR.Min	65 to 75	Maximum dorsiflexion at any point in the pedal stroke defined by the knee-ankle line and the heel–foot-line.
Ankle Maximum	AR.Max	90 to 100	Maximum plantarflexion at any point in the pedal stroke defined by the knee–ankle line and the heel-foot-line.
Ankle Range	AR	20 to 30	The difference between ankle maximum and ankle minimum.
Ankle Angle at Bottom	AAB	90 to 100	The ankle angle at the bottom of the pedal stroke (180°).
Maximum Knee Flexion	MKF	107 to 113	Maximum flexion of the knee joint at any point in the pedal stroke defined by the hip–knee line and the knee–ankle line
Maximum Knee Extension	MKE	32 to 42	Maximum extension of the knee joint at any point in the pedal stroke defined by the hip–knee line and the knee–ankle line
Knee Angle Range	KAR	70 to 75	The difference between knee angle flexion and knee angle extension.
Knee Forward of Foot	KFF	−10 to 10	The fore and aft offset of the knee marker relative to the foot marker captured at the forward part of the pedal stroke (3 o’clock or 90° down). A negative number indicates a knee that is aft of neutral.
Kee Forward of Spindle	KFS	−35 to −5	The fore and aft offset of the knee marker relative to the pedal spindle at 3 o’clock in the pedal stroke (90° in the downstroke).
Knee Travel Tilt	KTT	−2 to 4	The frontal plane angle of the tracing created by the moving knee marker with respect to vertical. A positive number indicates a knee that tracks away from the bike in the upstroke. A negative number represents a knee that tracks towards the bike in the upstroke. See the front view of the knee path for visual representation of this measurement.
Knee Lateral Travel	KLT	5 to 36	The magnitude of the lateral movement of the knee
Hip Angle Closed	HAC	66 to 76	The most closed angle of the hip joint defined by the knee, hip and shoulder marker.
Hip Angle Open	HAO	110 to 120	The most open angle of the hip joint defined by the knee, hip and shoulder marker.
Hip Angle Range	HAR	40 to 45	The difference between hip angle open and closed.
Hip Lateral Travel	HLT	5 to 20	The magnitude of the lateral movement of the hip
Back Angle	BA	50 to 65	The angle of the back relative to the horizon defined by the hip and shoulder marker
Shoulder Angle to Wrist	SAW	65 to 75	The angle of the shoulder joint defined by the hip, shoulder, and wrist markers.
Shoulder Angle to Elbow	SAE	60 to 70	The angle of the shoulder joint defined by the hip, shoulder, and elbow markers.

**Table 3:** Descriptive and inferential analysis of all kinematic variables.

Measurements		Retul		Vicon		Mean Differences					Correlations		SEM			BA	
		M	SD	M	SD	MD	SD	CI95%		*p*	ICC (CI95%)	*r*	Value	ICC (CI95%)		*t*	Reg
**A.Min**	**Ankle Minimum**	72.82	2.60	72.64	3.11	0.18	1.83	−1.05	1.41	0.75	0.894 (0.601 to 0.972)	0.808	0.822	74.43	71.21	0.85	0.36
**A.Max**	**Ankle Maximum**	94.82	3.49	94.64	3.64	0.18	1.83	−1.05	1.41	0.75	0.935 (0.755 to 0.982)	0.868	1.103	96.98	92.66	0.90	0.34
**AR**	**Ankle Range**	22.82	2.96	22.64	3.29	0.18	1.83	−1.05	1.41	0.75	0.913 (0.674 to 0.977)	0.833	0.936	24.65	20.98	0.86	0.01
**AAB**	**Ankle Angle at Bottom**	94.46	3.24	94.09	3.36	0.36	2.01	−0.99	1.72	0.56	0.903 (0.643 to 0.974)	0.814	1.023	96.46	92.45	0.79	0.56
**MKF**	**Maximum Knee Flexion**	111.05	1.15	111.36	1.50	0.14	0.89	−0.60	1.49	0.93	0.911 (0.660 to 0.976)	0.823	0.364	111.76	110.34	0.51	0.66
**MKE**	**Maximum Knee Extension**	41.46	1.86	41.18	1.94	0.27	1.27	−0.58	1.13	0.49	0.879 (0.561 to 0.967)	0.777	0.589	42.61	40.30	0.75	0.55
**KAR**	**Knee Angle Range**	69.73	4.03	69.64	3.96	0.09	2.17	−1.36	1.55	0.89	0.927 (0.724 to 0.980)	0.853	1.274	72.22	67.23	0.95	0.01
**HAC**	**Hip Angle Closed**	48.55	3.72	48.36	4.18	0.18	2.56	−1.54	1.90	0.82	0.892 (0.589 to 0.971)	0.796	1.178	50.85	46.24	0.91	0.96
**HAO**	**Hip Angle Open**	92.91	5.91	93.00	6.51	−0.09	2.70	−1.90	1.72	0.91	0.955 (0.830 to 0.988)	0.910	1.868	96.57	89.25	0.97	0.54
**HAR**	**Hip Angle Range**	44.09	1.70	43.46	3.05	0.64	2.06	−0.75	2.02	0.33	0.787 (0.251 to 0.942)	0.764	0.538	45.14	43.04	0.63	0.81
**BA**	**Back Angle**	23.64	3.23	23.46	3.98	0.18	2.09	−1.22	1.59	0.78	0.917 (0.686 to 0.978)	0.853	1.022	25.64	21.63	0.86	0.09
**SAW**	**Shoulder Angle to Wrist**	112.18	8.75	112.46	8.31	−0.27	3.80	−2.82	2.28	0.82	0.952 (0.821 to 0.987)	0.902	2.767	117.61	106.76	0.92	0.10
**SAE**	**Shoulder Angle to Elbow**	75.46	4.68	75.18	5.71	0.27	2.33	−1.29	1.84	0.71	0.951 (0.820 to 0.987)	0.918	1.479	78.35	72.56	0.89	0.30
**KFF**	**Knee Forward of Foot**	64.18	5.47	64.00	16.91	0.18	5.60	−3.58	3.94	0.92	0.972 (0.895 to 0.992)	0.944	1.730	67.57	60.79	0.97	0.08
**KFS**	**Knee Forward of Spindle**	67.55	4.95	66.73	18.01	0.82	5.86	−3.12	4.76	0.65	0.970 (0.989 to 0.992)	0.954	1.565	70.61	64.48	0.50	0.44
**KTT**	**Knee Travel Tilt**	3.64	1.12	3.55	1.92	0.09	1.30	−0.78	0.96	0.82	0.807 (0.250 to 0.949)	0.754	0.354	4.33	2.94	0.85	0.55
**KLT**	**Knee Lateral Travel**	27.46	6.79	27.27	5.78	0.18	4.26	−2.68	3.05	0.89	0.881 (0.545 to 0.968)	0.781	2.146	31.66	23.25	0.92	0.05
**HLT**	**Hip Lateral Travel**	16.55	5.35	16.55	5.80	0.00	2.41	−1.62	1.62	1.00	0.955 (0.832 to 0.988)	0.910	1.693	19.86	13.23	1.00	0.03

Mean (M); Standard deviation (SD); Mean difference between groups (MD); Confidence interval 95% (CI95%); Intraclass correlation coefficient (ICC); Pearson correlation (*r*); Standard error measurements (SEM); Bland-Altman analysis (BA); *t* (Student *t*-test for the measurement of differences between systems); Linear regression of Bland–Altman analysis (Reg); Student *t*-test statistical difference between means (*p*); Significance level (*p* < 0.05).

## Data Availability

Additional data from this study were not allowed to be shared by participant institutions.

## Supplementary Materials

The Supplementary Materials are available online at https://www.mdpi.com/article/10.3390/s21134473/s1.
