# Supplementary material for: Validation of a 3D Camera System for Cycling Analysis
Source: Sensors (Basel). 2021 Jun 30;21(13):4473. doi: 10.3390/s21134473 (PMC8271997; doi:10.3390/s21134473)
Supplement: Supplementary file 1 [file sensors-21-04473-s001.zip › Bland_Altman_Plots.pdf]

Bland-Altman Plot 01: A.Min Variable

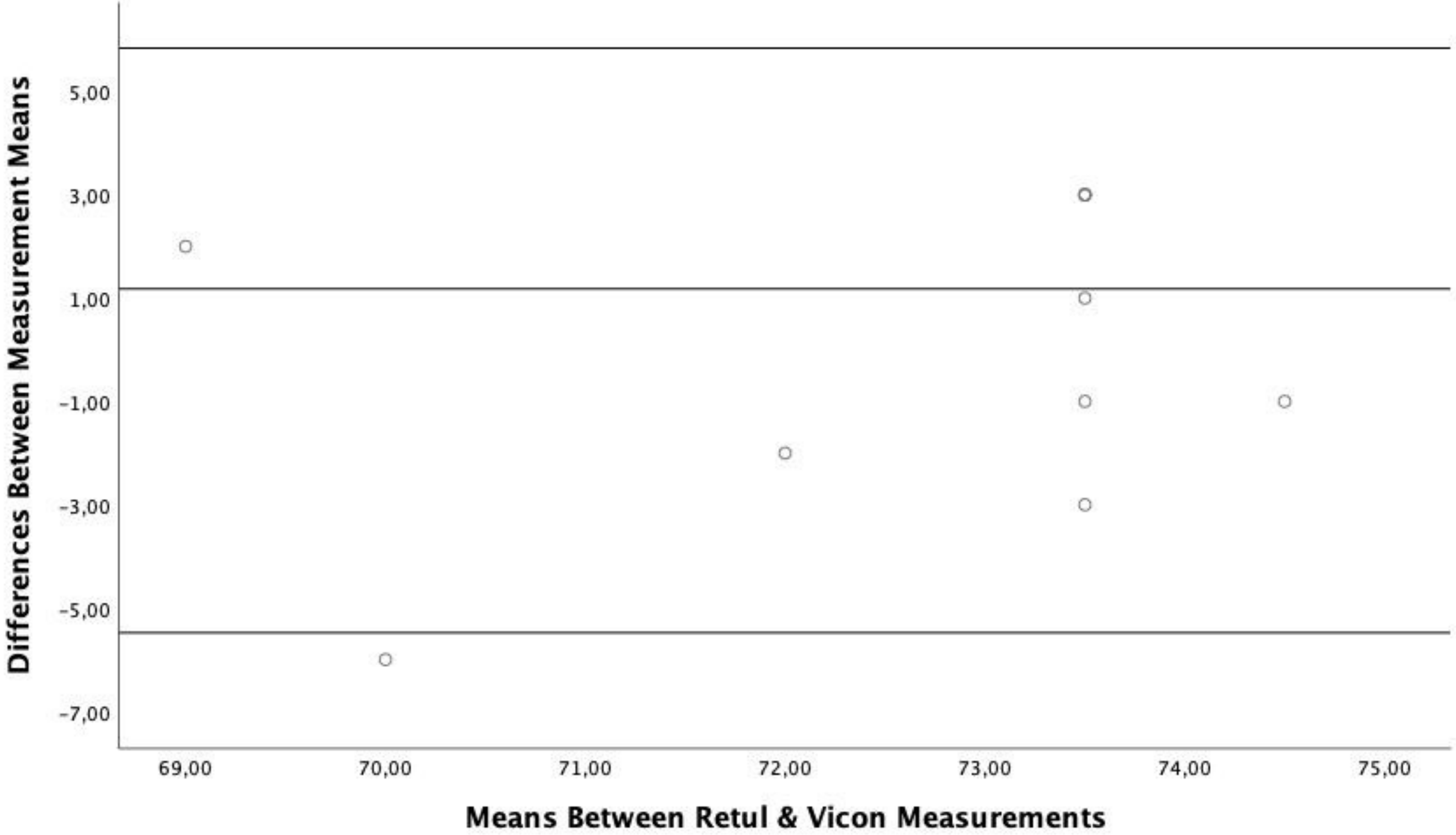

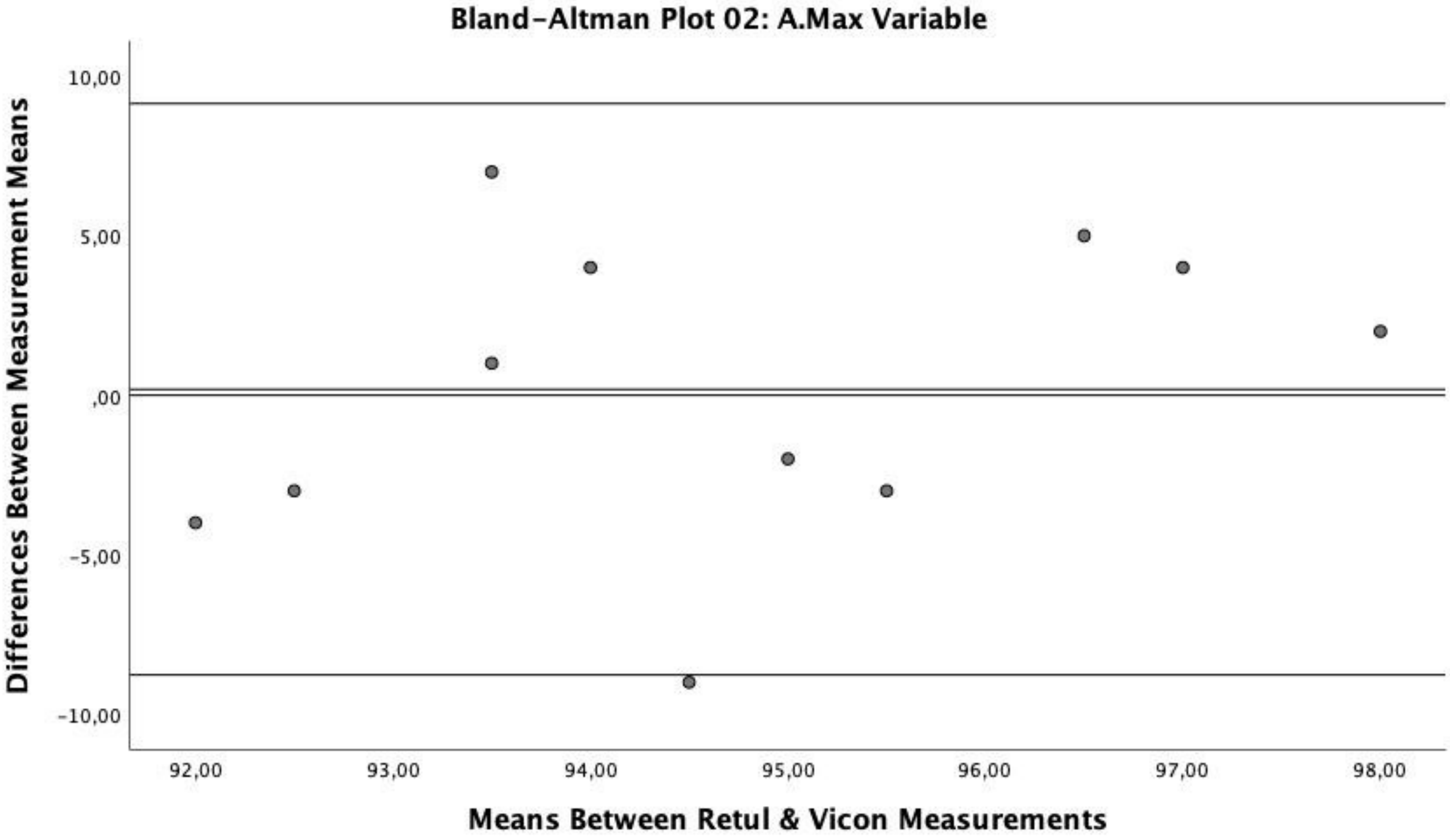
Bland-Altman Plot 02: A.Max Variable
Differences Between Measurement Means
10,00
5,00
,00
-5,00
-10,00
92,00
93,00
94,00
95,00
96,00
97,00
98,00
Means Between Retul & Vicon Measurements

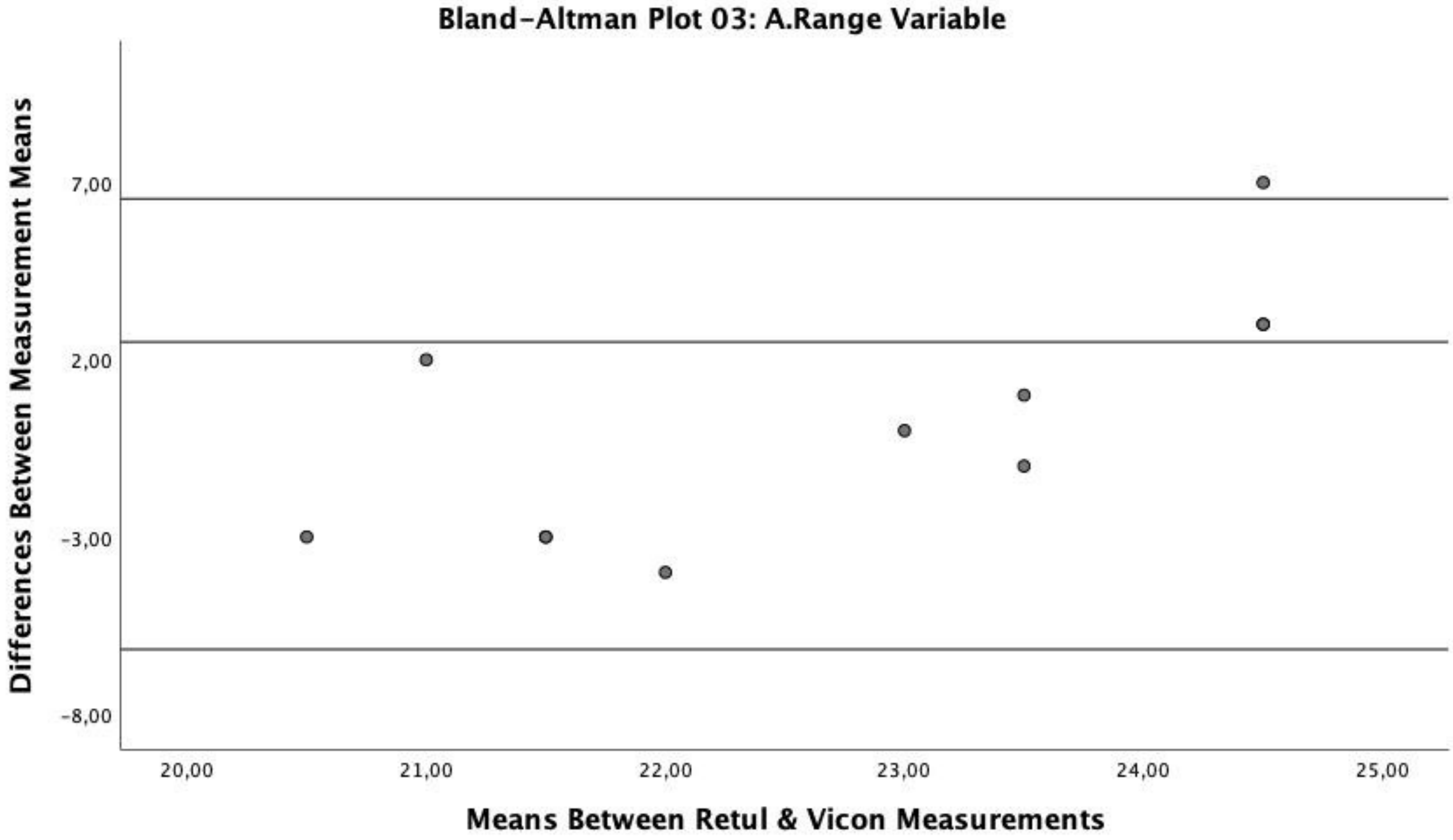
Bland-Altman Plot 03: A.Range Variable
Differences Between Measurement Means
7,00
2,00
-3,00
-8,00
20,00
21,00
22,00
23,00
24,00
25,00
Means Between Retul & Vicon Measurements

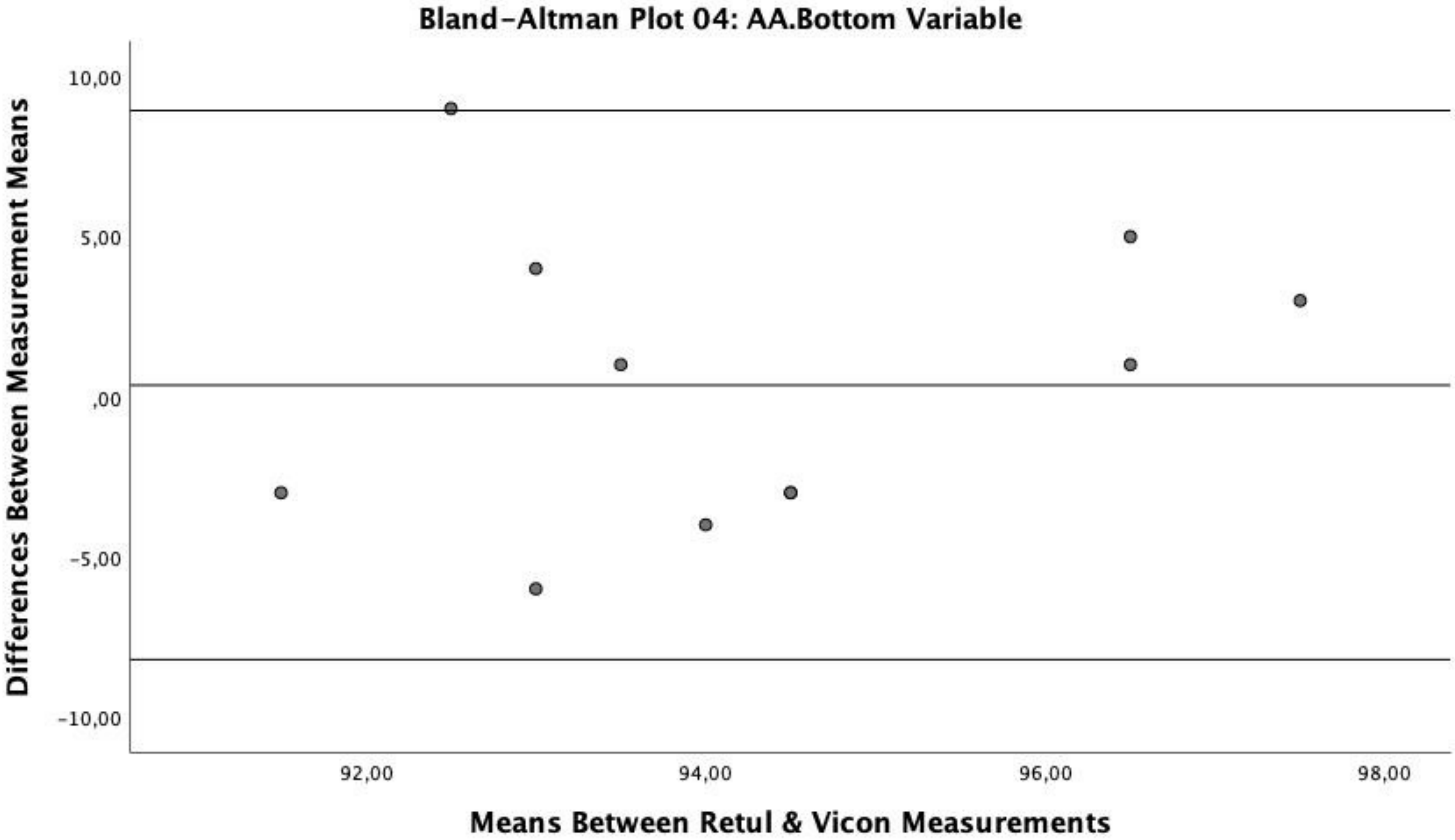
Bland-Altman Plot 04: AA.Bottom Variable
Differences Between Measurement Means
10,00
5,00
,00
-5,00
-10,00
92,00
94,00
96,00
98,00
Means Between Retul & Vicon Measurements

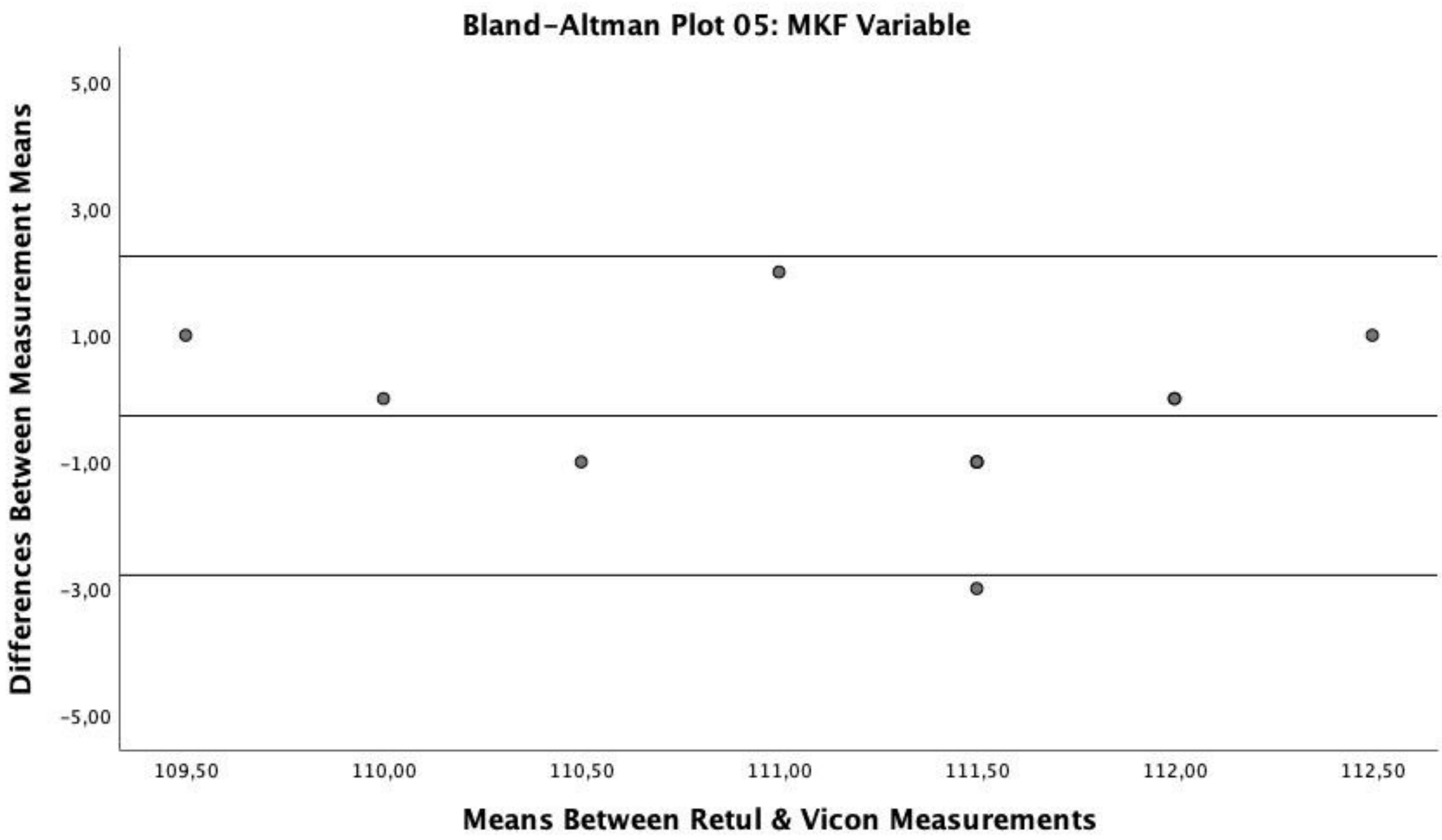

Bland-Altman Plot 05: MKF Variable
Differences Between Measurement Means
5,00
3,00
1,00
-1,00
-3,00
-5,00
109,50
110,00
110,50
111,00
111,50
112,00
112,50
Means Between Retul & Vicon Measurements

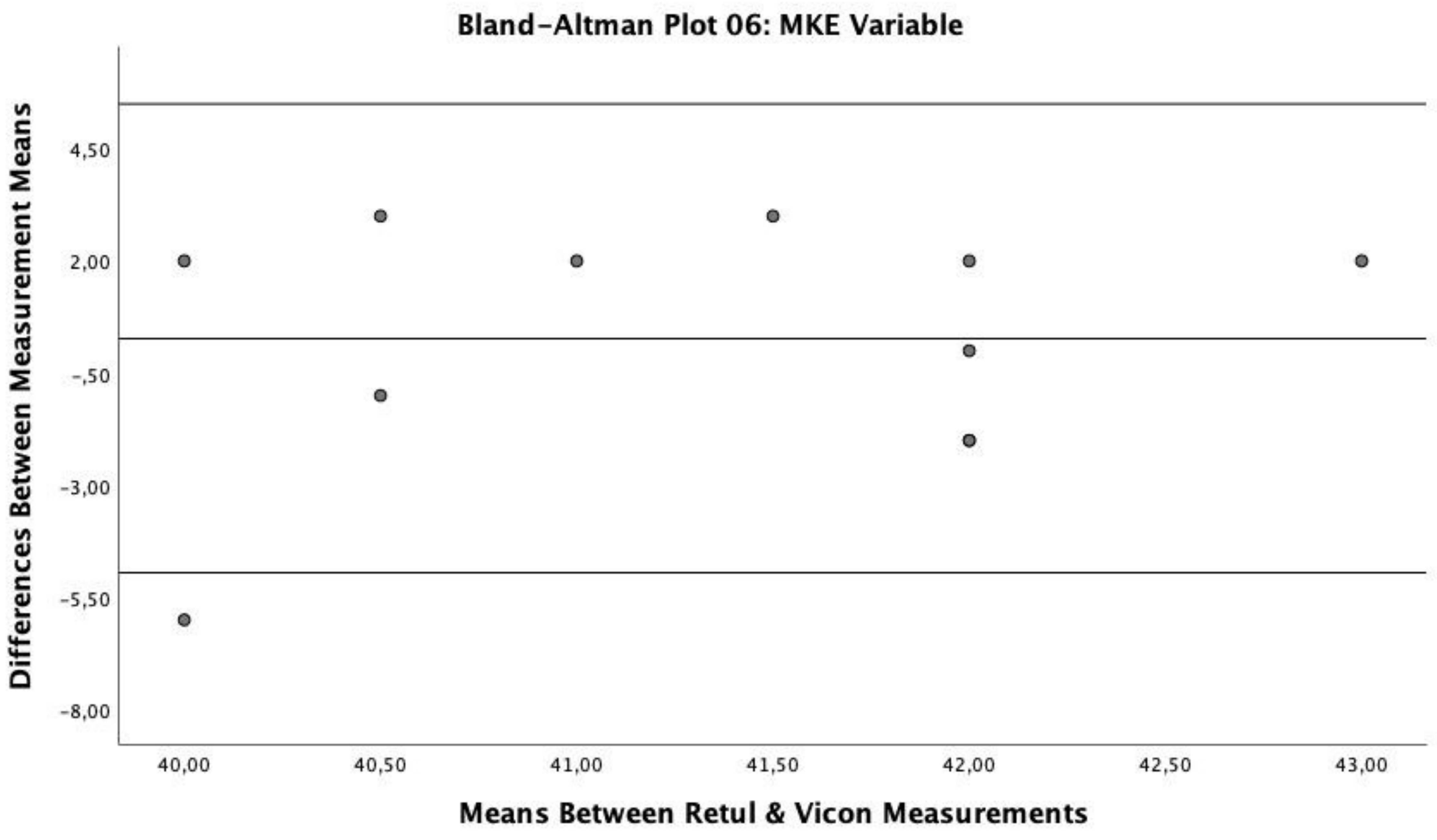

Bland–Altman Plot 06: MKE Variable
Differences Between Measurement Means
4,50
2,00
-,50
-3,00
-5,50
-8,00
40,00
40,50
41,00
41,50
42,00
42,50
43,00
Means Between Retul & Vicon Measurements

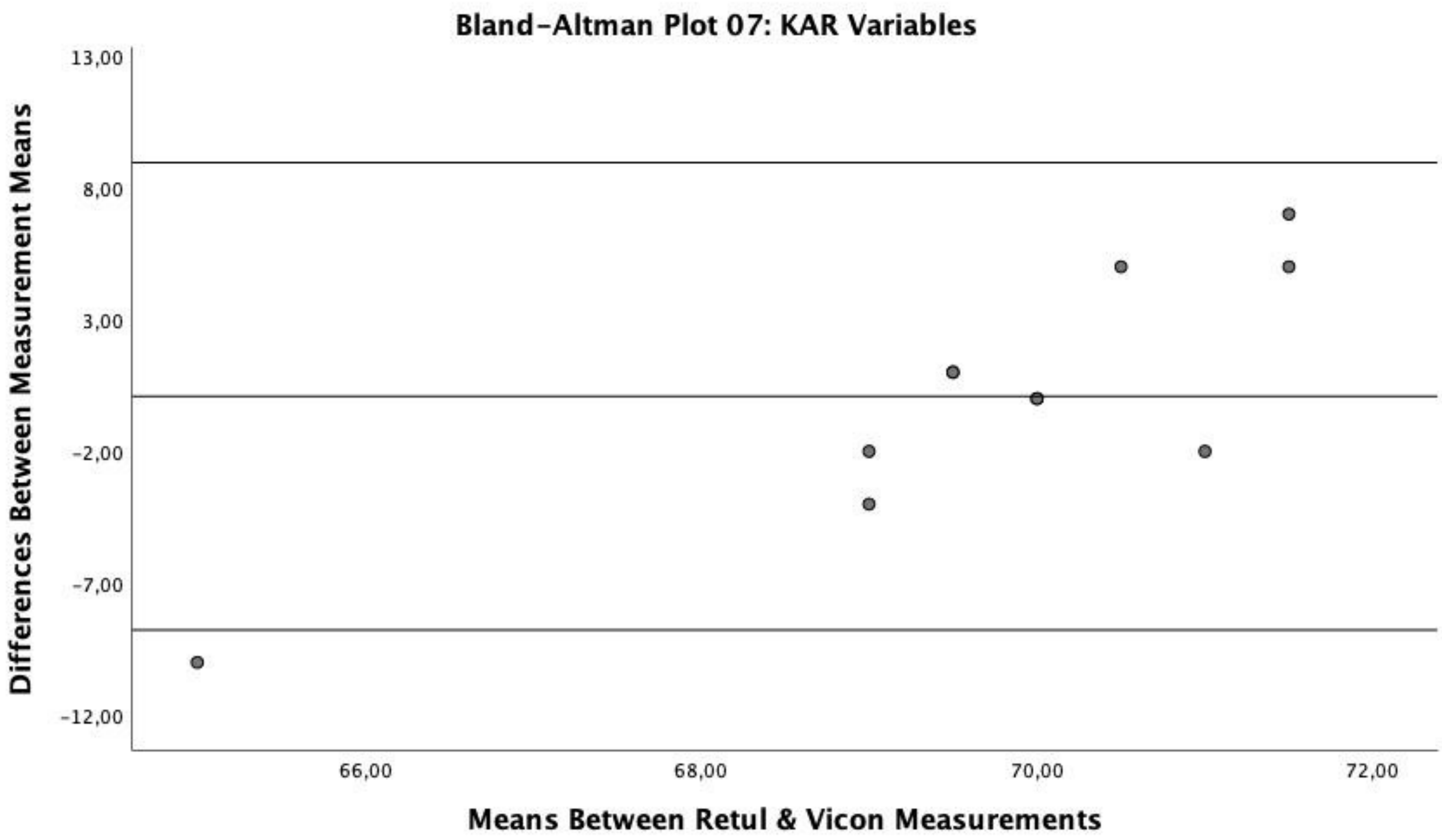
Bland-Altman Plot 07: KAR Variables
Differences Between Measurement Means
13,00
8,00
3,00
-2,00
-7,00
-12,00
66,00
68,00
70,00
72,00
Means Between Retul & Vicon Measurements

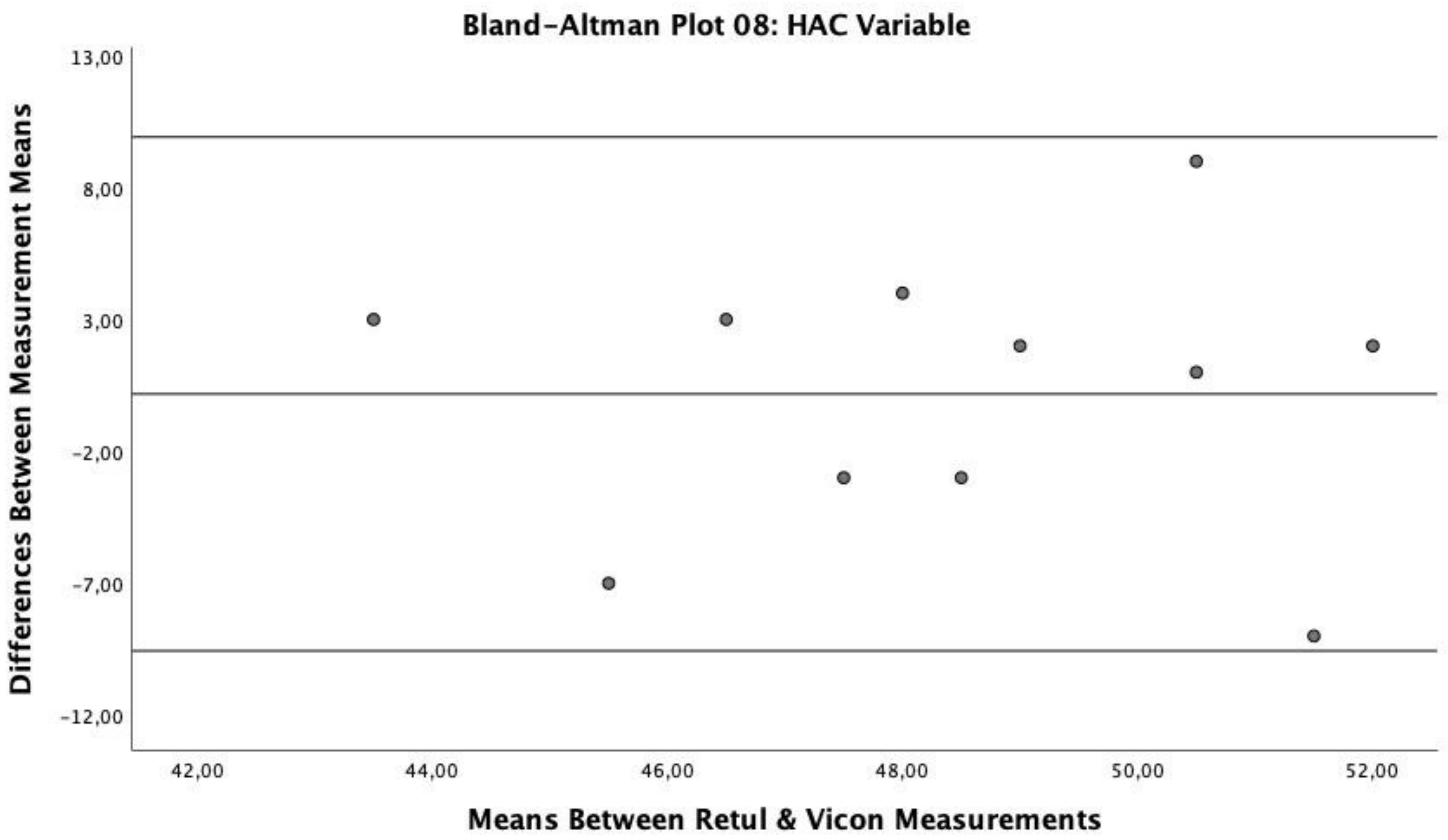
Bland-Altman Plot 08: HAC Variable
13,00
8,00
3,00
-2,00
-7,00
-12,00
Differences Between Measurement Means
42,00
44,00
46,00
48,00
50,00
52,00
Means Between Retul & Vicon Measurements

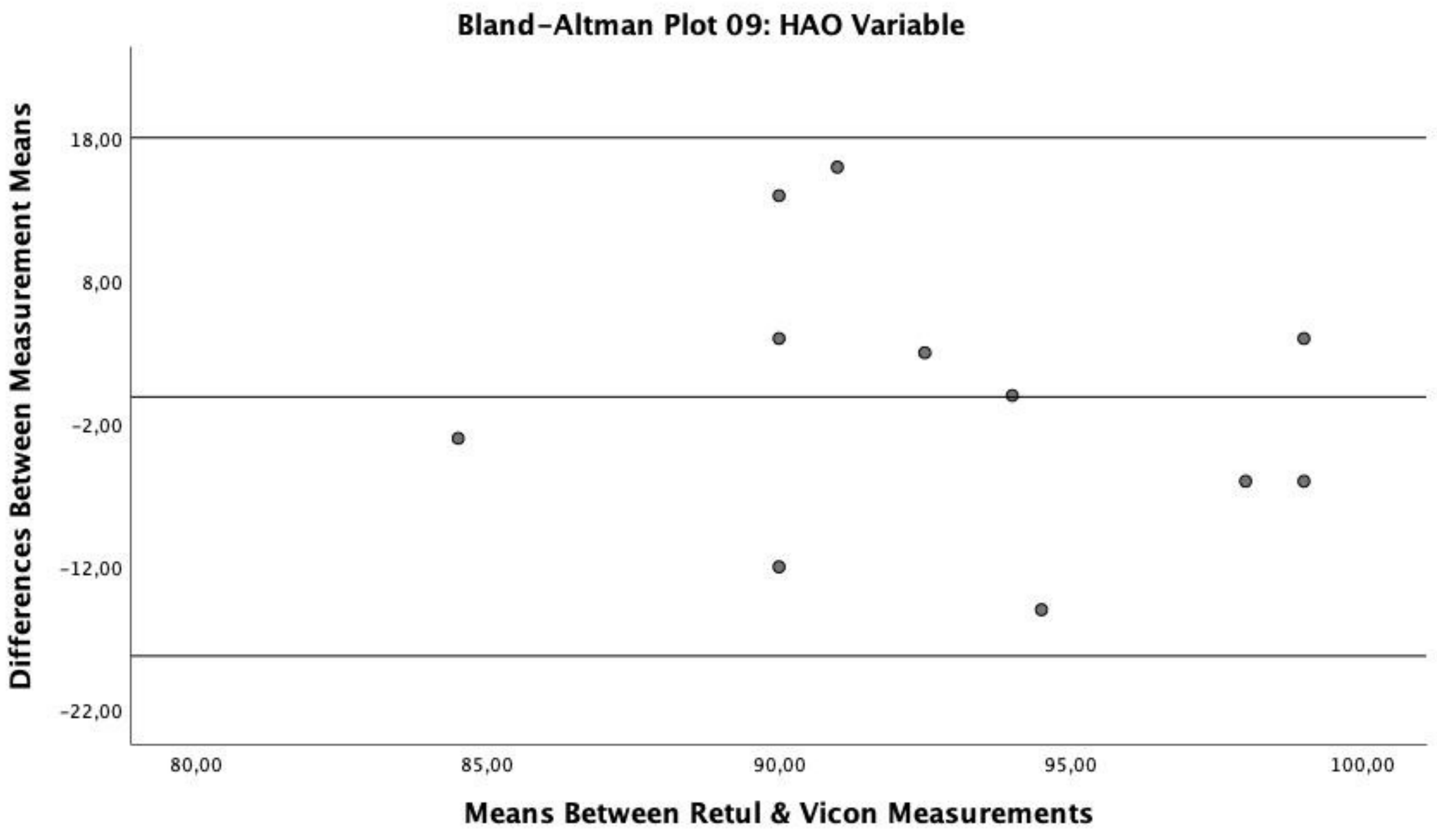
Bland-Altman Plot 09: HAO Variable
Differences Between Measurement Means
18,00
8,00
-2,00
-12,00
-22,00
80,00
85,00
90,00
95,00
100,00
Means Between Retul & Vicon Measurements

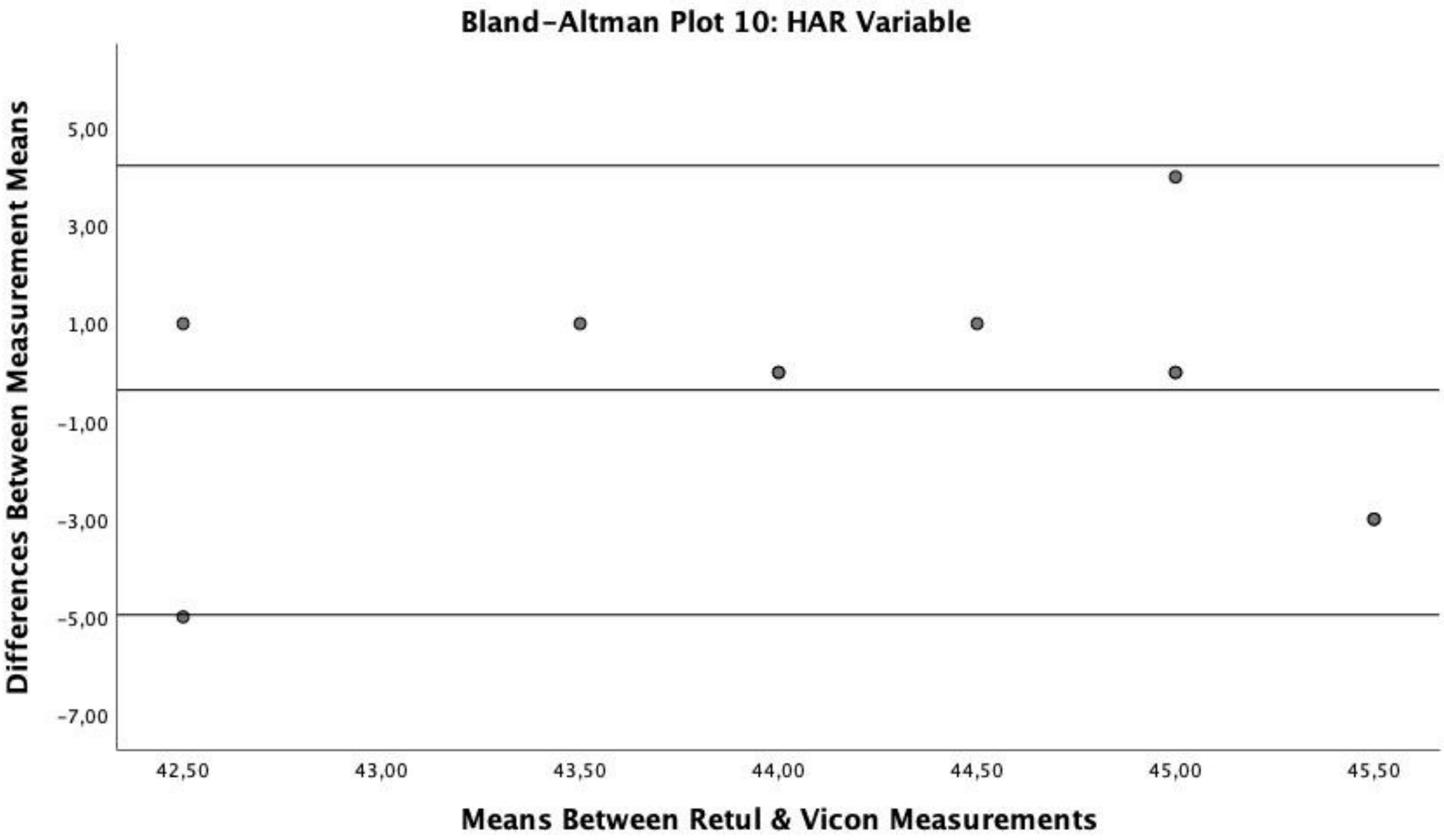
Bland-Altman Plot 10: HAR Variable
Differences Between Measurement Means
5,00
3,00
1,00
-1,00
-3,00
-5,00
-7,00
42,50
43,00
43,50
44,00
44,50
45,00
45,50
Means Between Retul & Vicon Measurements

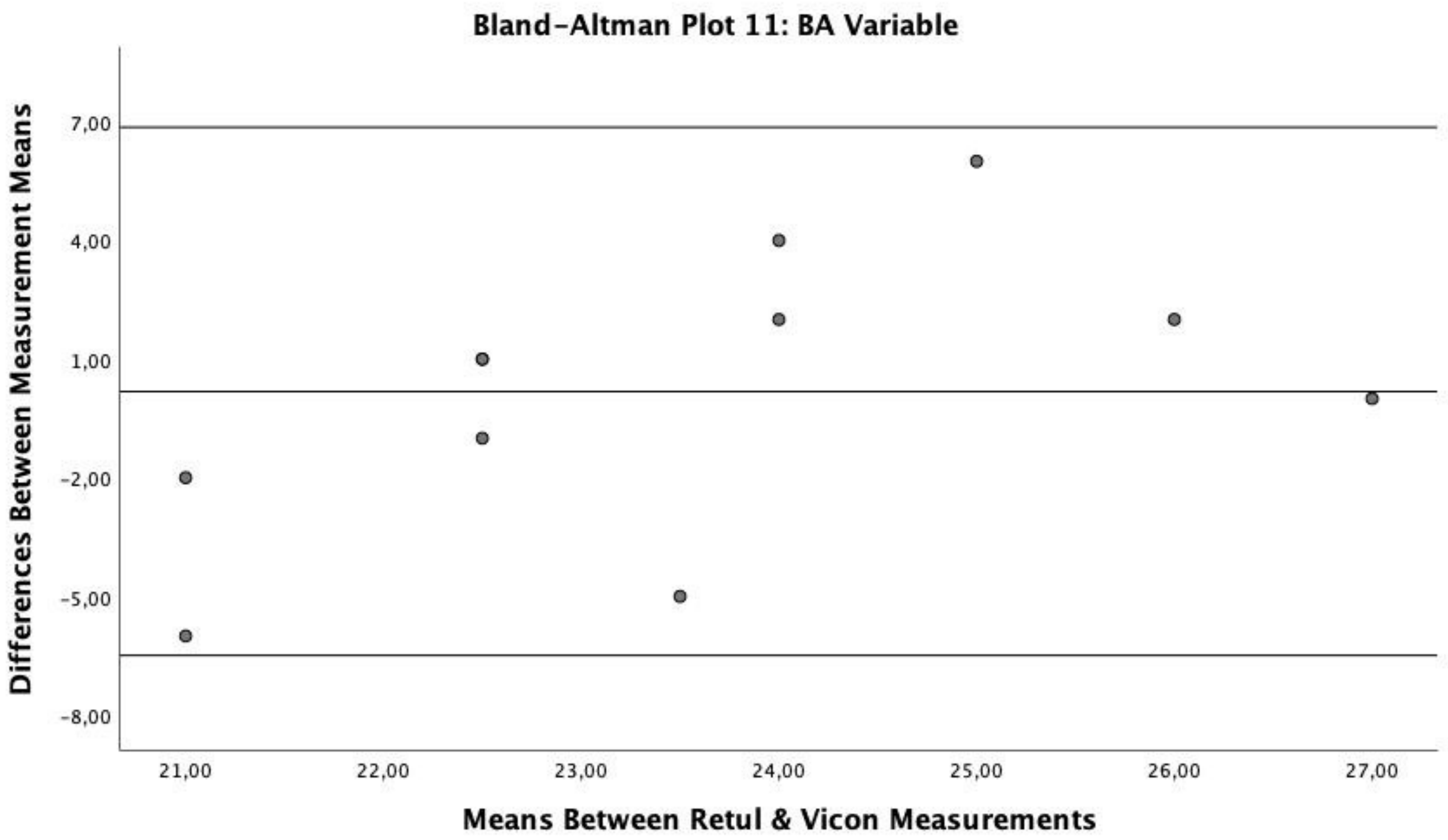
Bland-Altman Plot 11: BA Variable
Differences Between Measurement Means
7,00
4,00
1,00
-2,00
-5,00
-8,00
21,00
22,00
23,00
24,00
25,00
26,00
27,00
Means Between Retul & Vicon Measurements

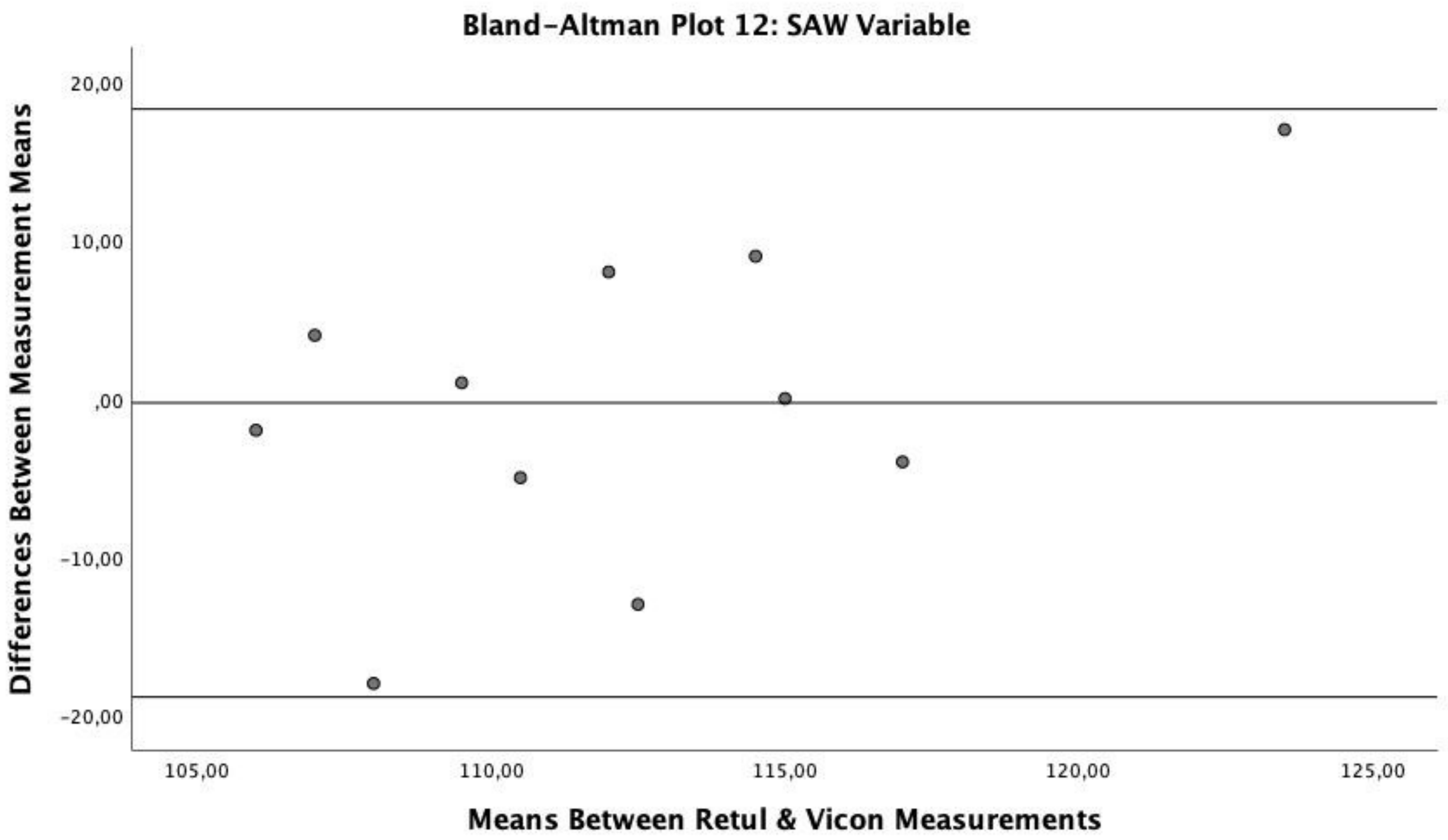
Bland-Altman Plot 12: SAW Variable
Differences Between Measurement Means
20,00
10,00
,00
-10,00
-20,00
105,00
110,00
115,00
120,00
125,00
Means Between Retul & Vicon Measurements

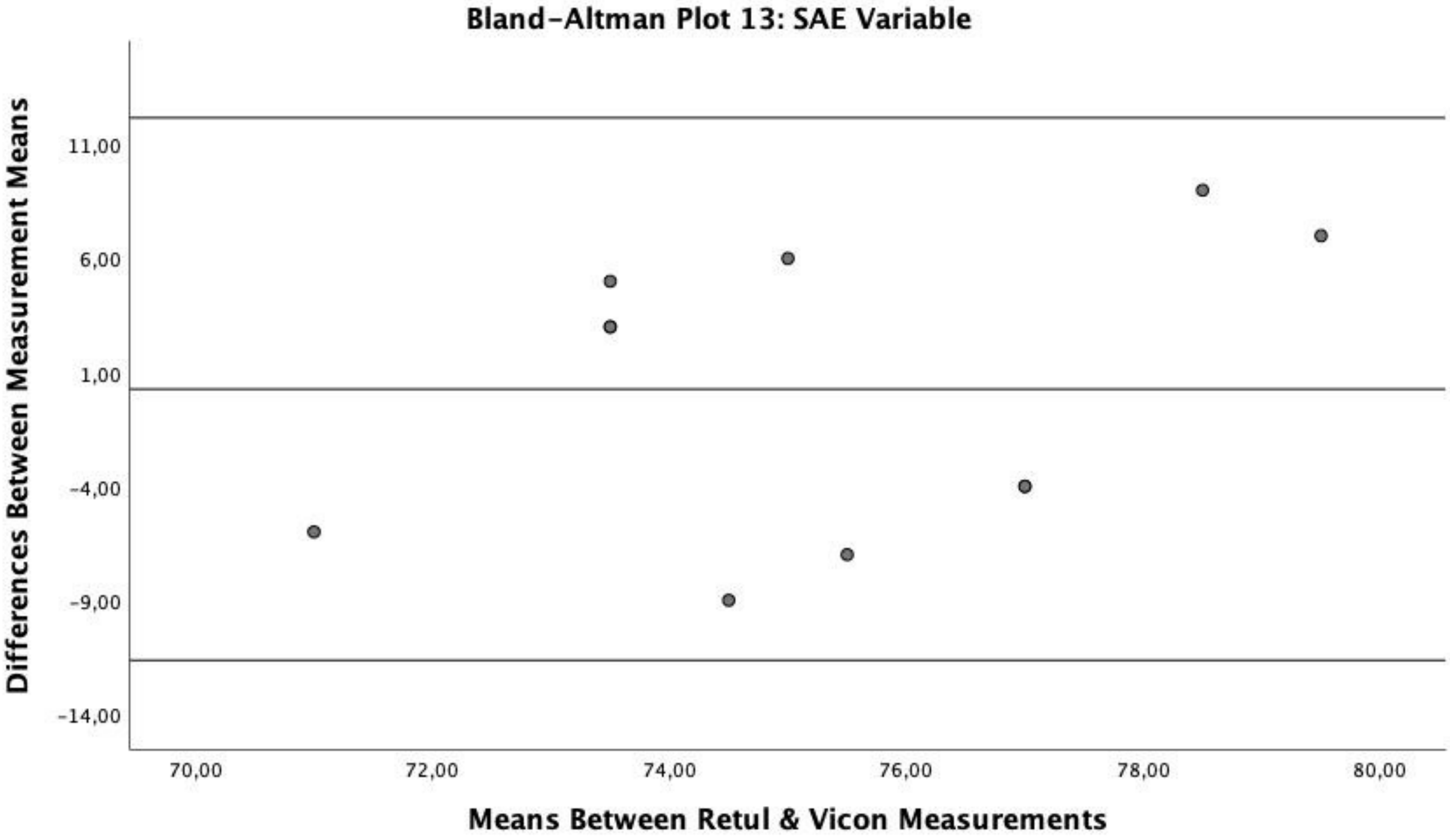

Bland-Altman Plot 13: SAE Variable
Differences Between Measurement Means
11,00
6,00
1,00
-4,00
-9,00
-14,00
70,00
72,00
74,00
76,00
78,00
80,00
Means Between Retul & Vicon Measurements

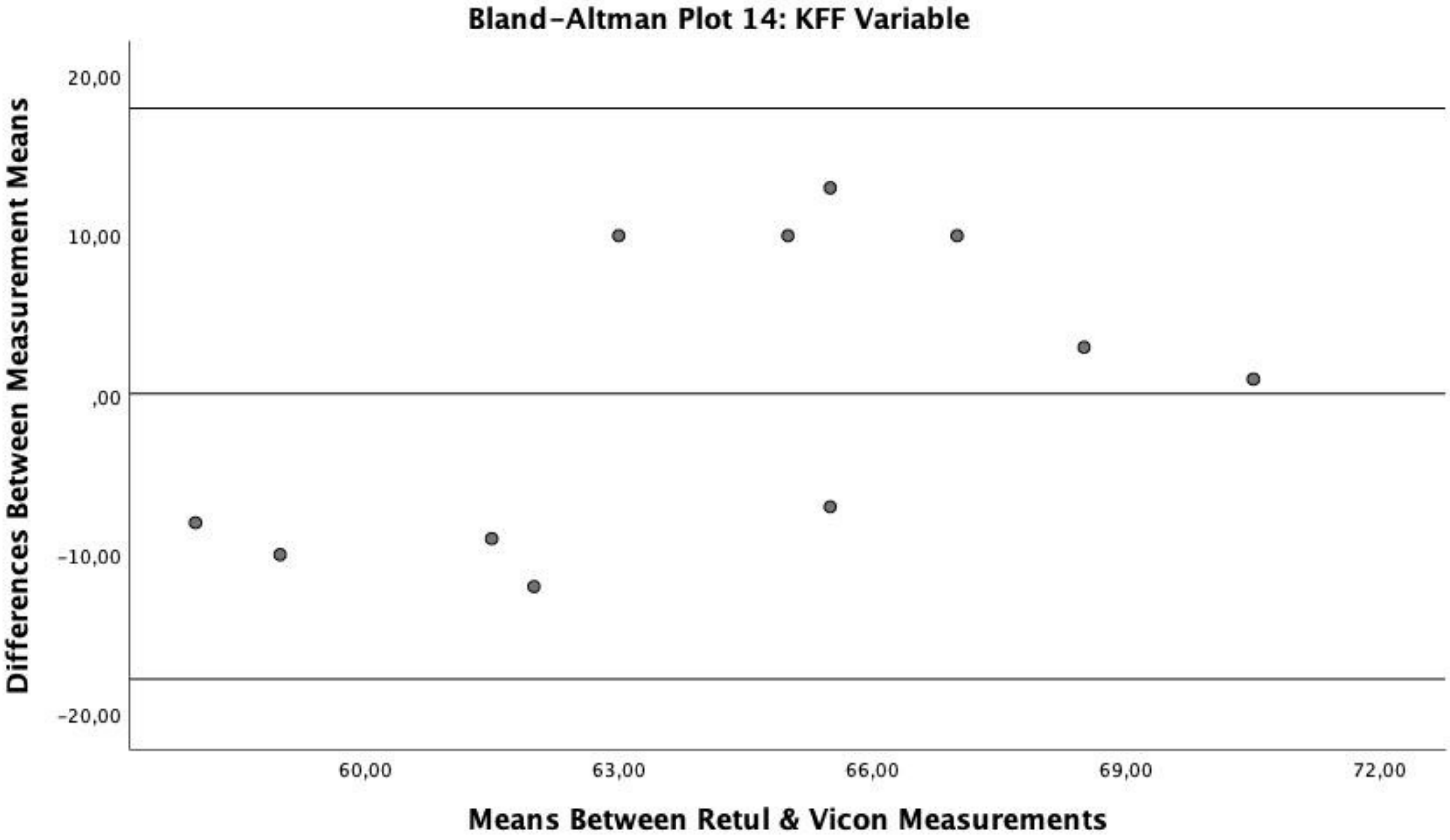
Bland-Altman Plot 14: KFF Variable
Differences Between Measurement Means
20,00
10,00
,00
-10,00
-20,00
60,00
63,00
66,00
69,00
72,00
Means Between Retul & Vicon Measurements

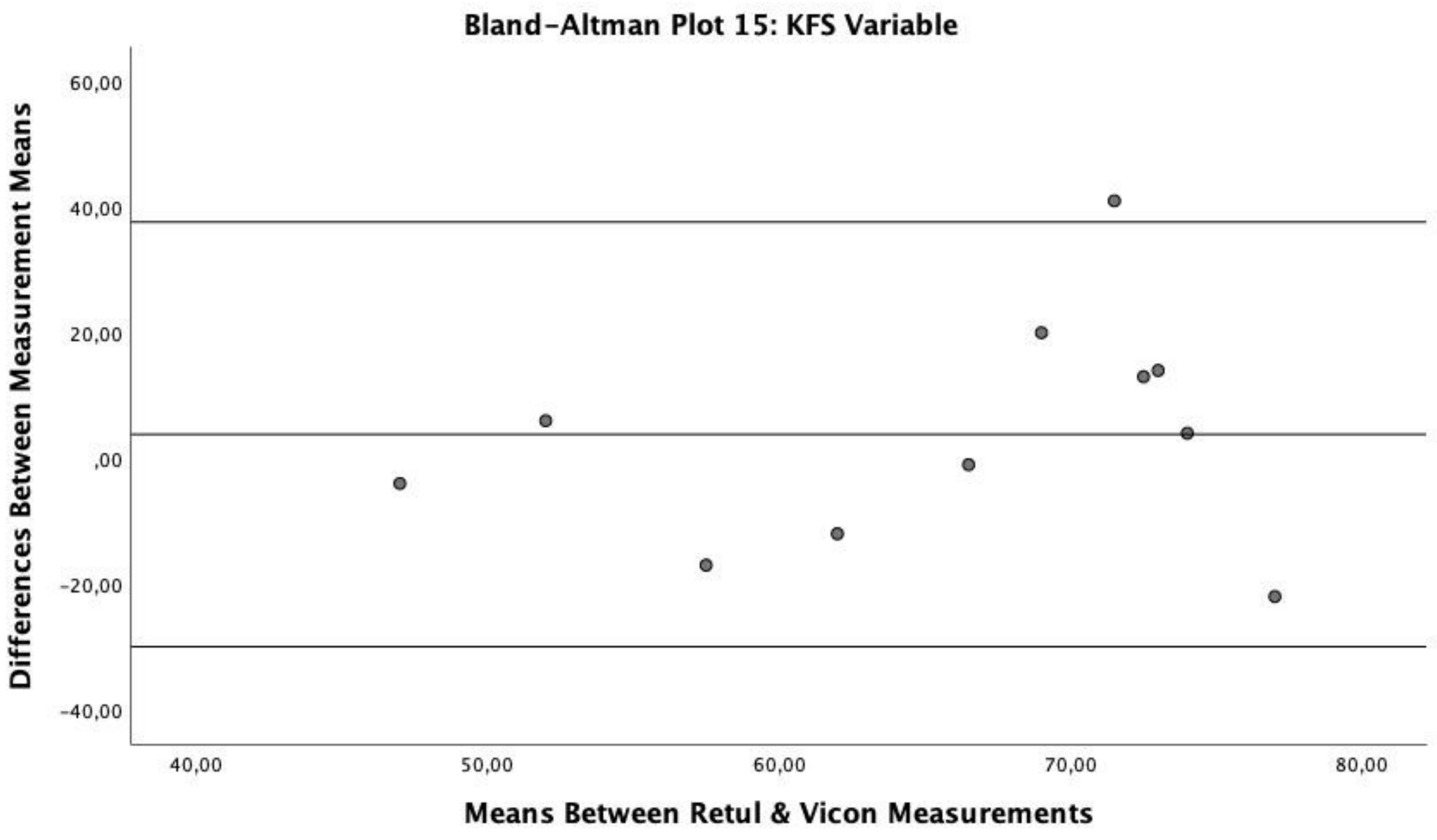
Bland-Altman Plot 15: KFS Variable
Differences Between Measurement Means
60,00
40,00
20,00
,00
-20,00
-40,00
40,00
50,00
60,00
70,00
80,00
Means Between Retul & Vicon Measurements

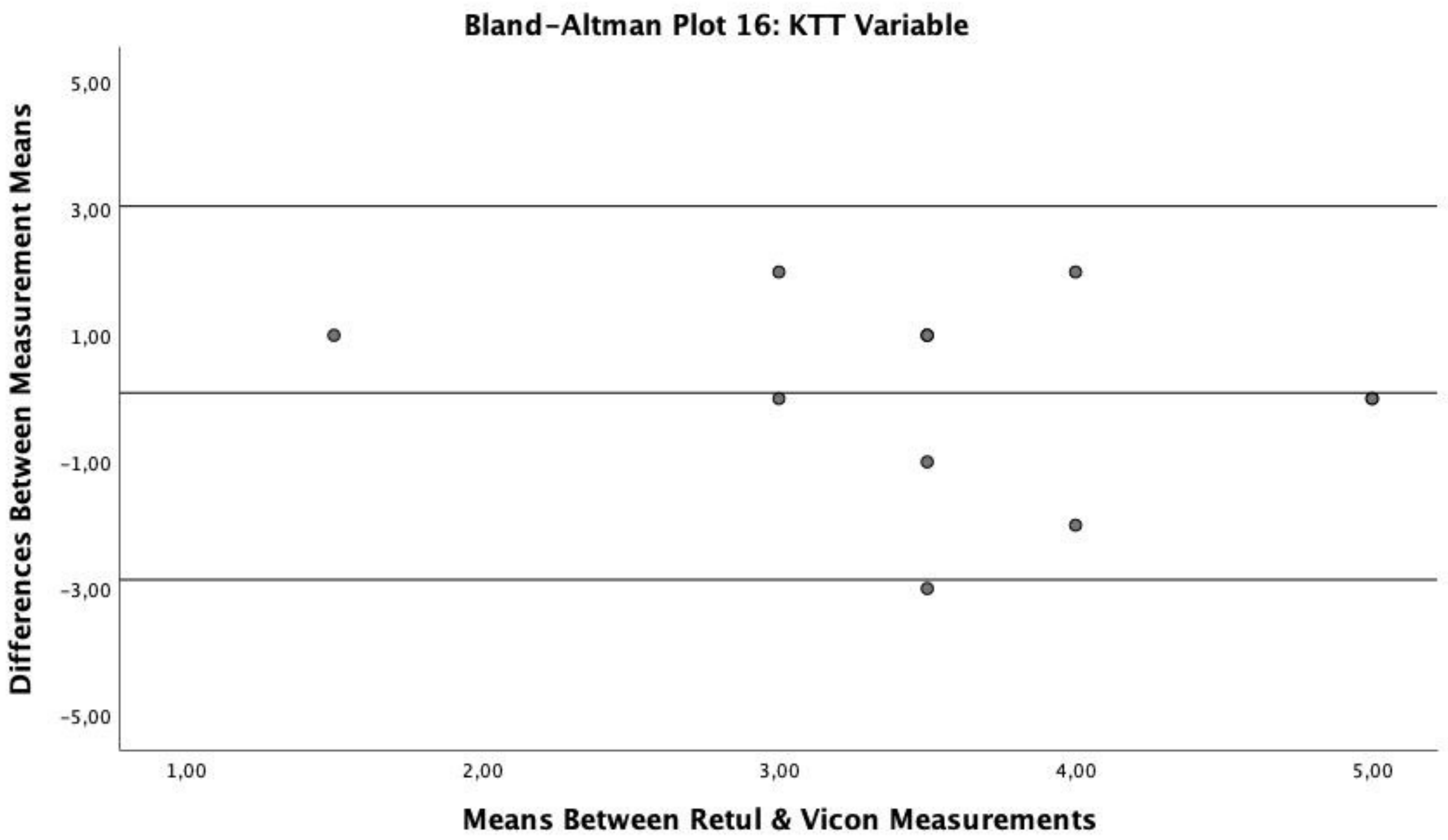
Bland-Altman Plot 16: KTT Variable
5,00
3,00
1,00
-1,00
-3,00
-5,00
Differences Between Measurement Means
1,00
2,00
3,00
4,00
5,00
Means Between Retul & Vicon Measurements

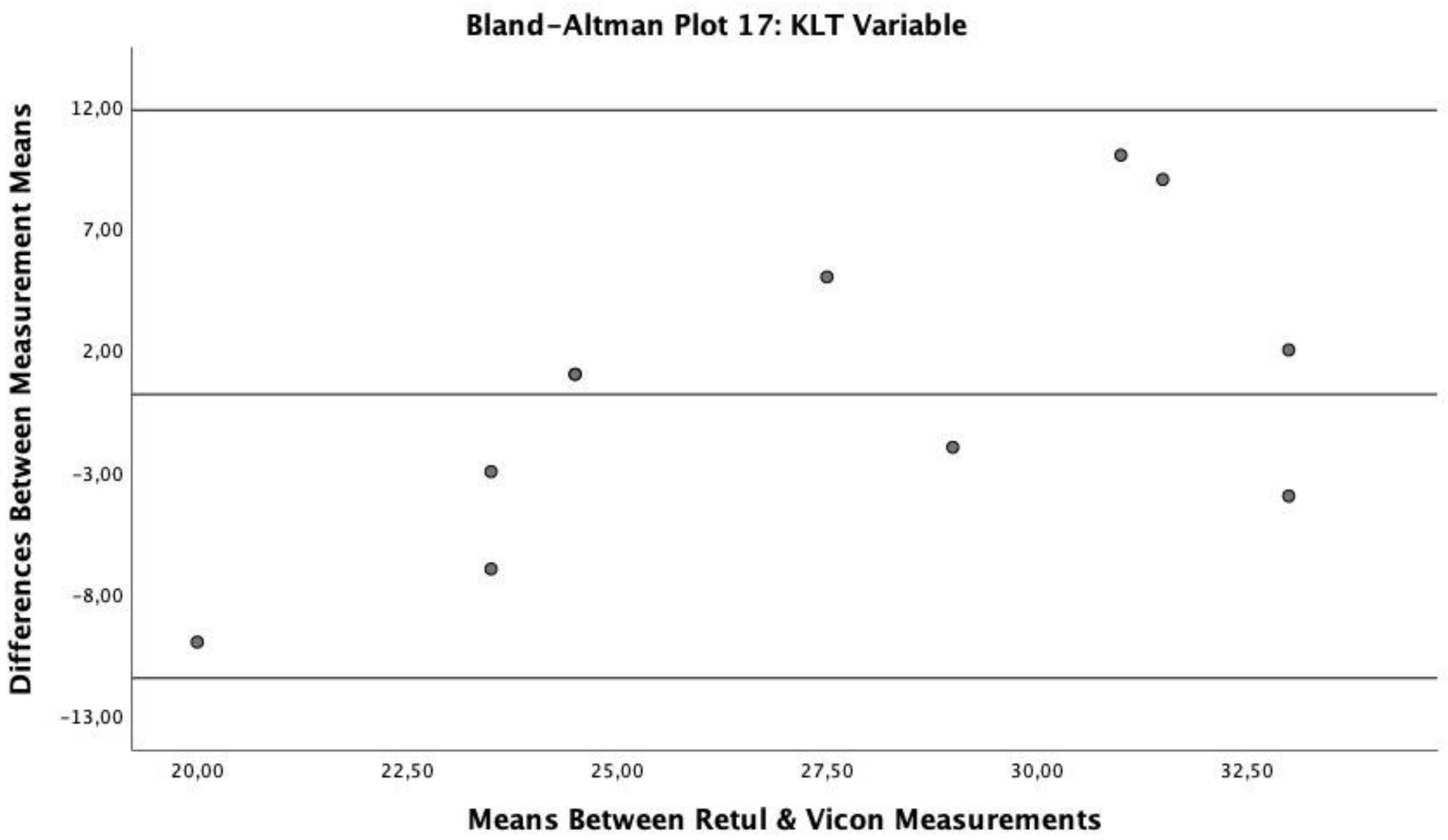
Bland-Altman Plot 17: KLT Variable
Differences Between Measurement Means
12,00
7,00
2,00
-3,00
-8,00
-13,00
20,00
22,50
25,00
27,50
30,00
32,50
Means Between Retul & Vicon Measurements

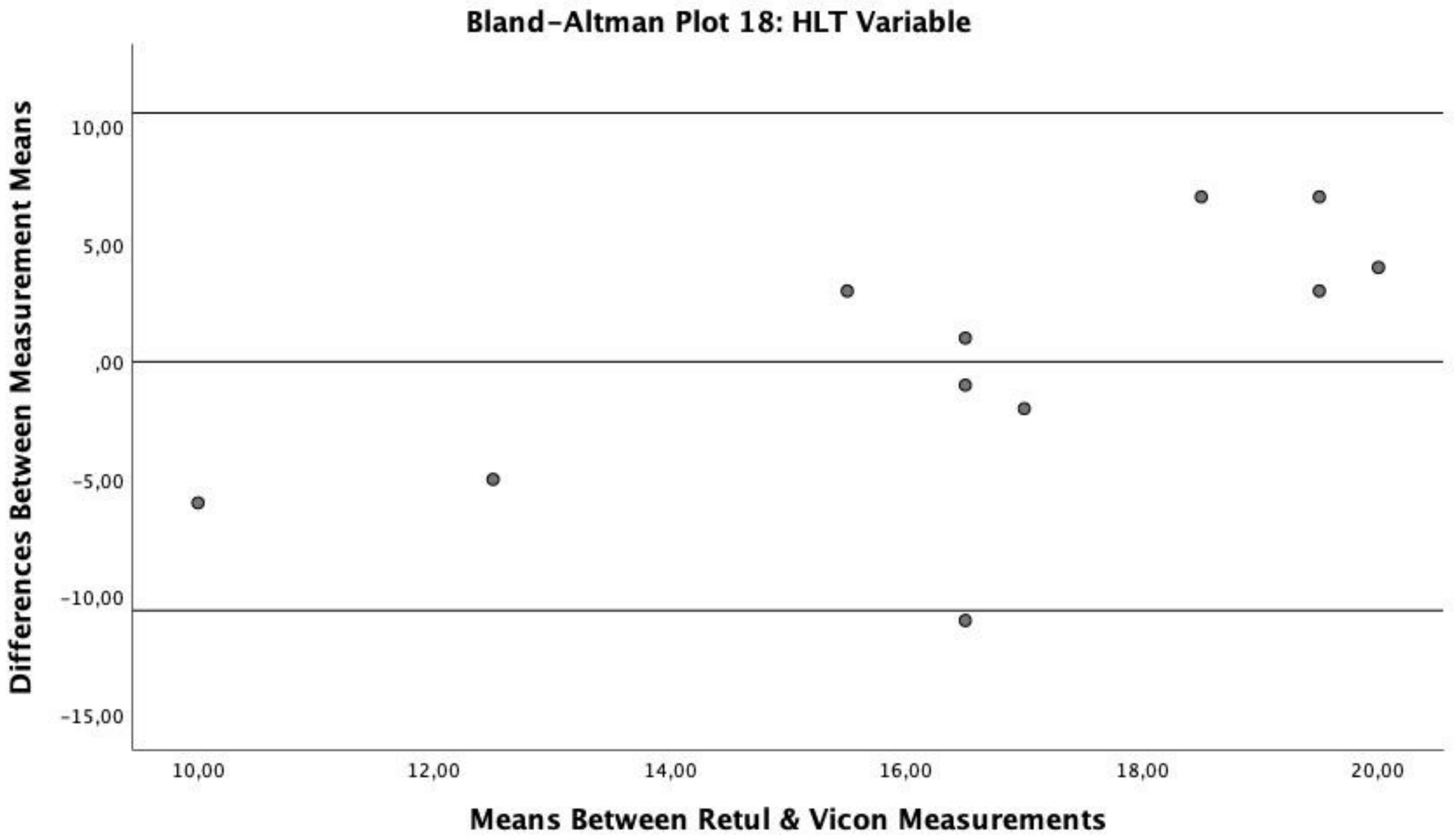
Bland-Altman Plot 18: HLT Variable
Differences Between Measurement Means
10,00
5,00
,00
-5,00
-10,00
-15,00
10,00
12,00
14,00
16,00
18,00
20,00
Means Between Retul & Vicon Measurements
