# Supplementary material for: Validation of a 3D Camera System for Cycling Analysis
Source: Sensors (Basel). 2021 Jun 30;21(13):4473. doi: 10.3390/s21134473 (PMC8271997; doi:10.3390/s21134473)
Supplement: Supplementary file 1 [file sensors-21-04473-s001.zip › Bonita_Camera_Specs.pdf]

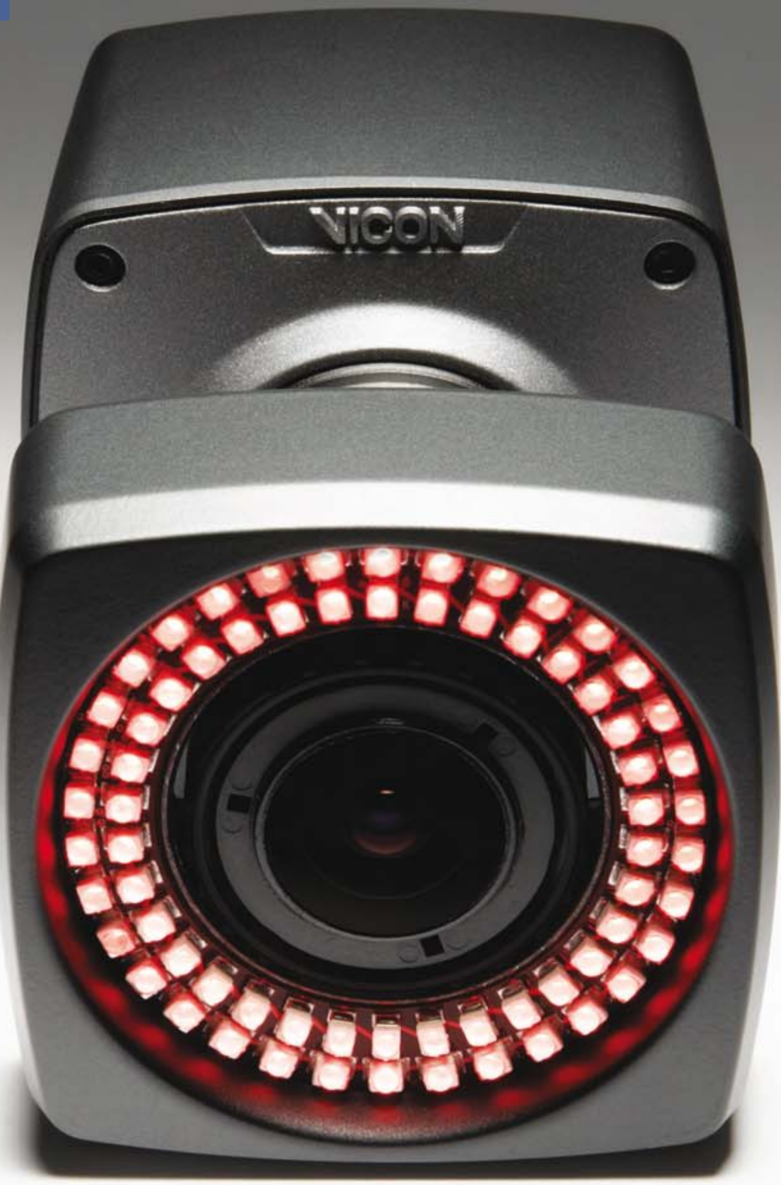

Bonita

Size.Performance.Reliability.

Pioneering motion capture since 1984.

Bonita

Size. Performance. Reliability.

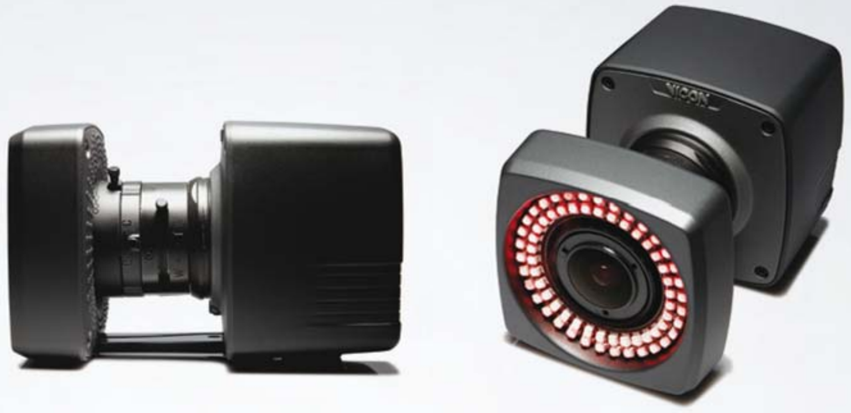

Specifications

All Bonita Range

Interface	Gigabit Ethernet, RJ45
System latency	2ms
Strobe	68 high powered NIR LEDs @ 780nm
Power	802.3af PoE

Bonita 3

Frame Rate	240 fps
Resolution	0.3 megapixel (640 x 480)
Lens Operating Range	Up to 12m
Angle of View Wide (4mm)	82.7° x 66.85°
Angle of View Narrow (12mm)	32.7° x 24.81°

Bonita 10

Frame Rate	250 fps
Resolution	1 megapixel (1024 x 1024)
Lens Operating Range	Up to 13m
Angle of View Wide (4mm)	70.29° x 70.29°
Angle of View Narrow (12mm)	26.41° x 26.41°

Welcome to Bonita. Vicon's small optical camera family reliably streams the precise motion of markers, whether they are attached to people, animals or machines. Building on over 30 years of innovation, Bonita's speed, flexibility and affordability redefine the possibilities of motion capture.

Solution

Ergonomics. Training. Injury prevention. Synthetic environments. Static facial capture. Immersive reality. Virtual prototyping. Simulation. Visualization. Animation. Gait analysis. Sports biomechanics. Animal studies. Rehabilitation. Virtual camera tracking.

Precise

With its high resolution, quality optics and sophisticated algorithms, Bonita's new 1 megapixel camera, the Bonita 10, captures with precision, down to 0.5mm in a 4m x 4m volume.

Affordable

You don't have to choose between performance and price with the new Bonita family. With complete systems starting at $10,000 / £7,500, Bonita could change your idea of motion capture.

Fast

Capture speeds of up to 250 fps with the Bonita 10 and 240 fps with the Bonita 3, means you can easily and accurately capture the movements of fast moving objects.

Flexible

The beauty of the compact Bonita camera Is you can mount them anywhere. With its Variable focal length lens and single cable ethernet connection, it's easy to optimize your set up. From the footwell of a car, to a complex virtual reality cave environment, you won't lose performance with Bonita.

Plug and play

Some motion capture systems have a reputation for being hard to set up. Not with Bonita. Just like its big brother T-series, Bonita uses a single cable to provide everything it needs – power, data and synchronisation. Calibrate and start streaming data in less than 2 minutes.

Discover More

facebook.com/vicon
twitter.com/_vicon
youtube.com/vicon100

vicon.com/system/bonita

info@vicon.com

Denver
T: +1 303.799.8686
Los Angeles
T: +1 310.306.6131

Oxford
T: +44 (0) 1865 261800
Singapore
T: +65 6400 3500

Specifications subject to change without notice. Vicon acknowledges all trademarks.
All images copyright Vicon Motion Systems 2014.
