# Supplementary material for: Validation of a 3D Camera System for Cycling Analysis
Source: Sensors (Basel). 2021 Jun 30;21(13):4473. doi: 10.3390/s21134473 (PMC8271997; doi:10.3390/s21134473)
Supplement: Supplementary file 1 [file sensors-21-04473-s001.zip › Vantage_Camera_Specs.pdf]

Retül Vantage Technical Specifications

Overview:

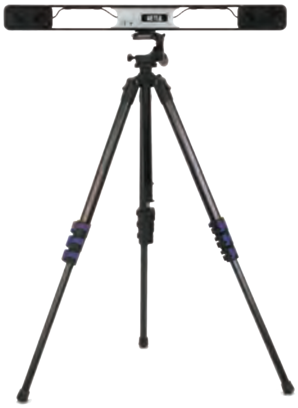

The Retül Vantage 3D motion capture hardware is a marker based optical tracking system. It tracks Infrared (IR) light emitted by light-emitting-diodes (LEDs). The 4 glass filters on the front of the unit triangulate the position of the LEDs in 3D space.

Accuracy & Speed:

The system individually tracks up to 15 markers without any loss of data rate. It currently tracks 8 markers on the cyclist's body and 4 markers on the bike measuring tool. Each of these LED markers can be located in 3D space at an accuracy of 0.4 mm (.016 in) RMS (average) and 1.0 mm (.039 in) Maximum error. The system captures all 8 cyclist markers and delivers 3D coordinates, with timestamps, 18 times per second (18 Hz).

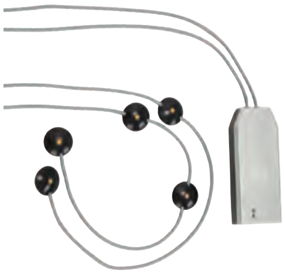

Field of View:

The system has an angular field of view. The further you are from the Vantage sensor, the wider and taller it can see. The calibration range of operation is between 1.5 m (4.9 ft) and 3.5 m (11.5 ft) of distance away from the sensor. Below are examples of the height and width of view at two sample distances from the sensor:
FOV @ 2 m (6.6 ft) range = 1.6 m (5.4 ft) wide x 2.1 m (6.9 ft) tall

Optimal System Layout:

The optimal operational layout of the system has the rider's bike in the center of the 0.9 m (3 ft) x 1.8 m (6 ft) rotating platform. The platform needs a 2.3 m (7.5 ft) diameter circle of clearance for it to rotate. The optimal distance from the knee marker to the Vantage sensor is 2.3 m (7.5 ft). This range number is displayed in real-time in the Retül software during the Run mode to assist with sensor positioning.

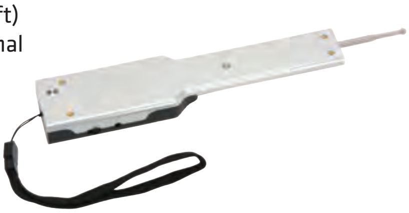

Key Technologies:

Synchronization:

The system has full control over all LED flashes and thus discreetly knows which LED is which. This eliminates all post processing work and allows the measurements and reports to be delivered automatically in seconds. This allows the fitter to use the technology as an aid to the fit process and not a distraction.

Stroke Intelligence:

During a Cyclist tracking recording, the software finds all the measurement events, creates a log of all body measurements, and averages all these measurements together for the total number of recorded strokes. This creates a true-to-life measure of the cyclist's movements and reduces the effects of atypical movements.

Cubic Time Interpolation:

Because of the timestamped 3D data that the Retül delivers, certain key motion events such as foot forward position and maximum leg extension can be interpolated to increase measurement accuracy. This allows the system to find these events even if a data capture didn't exactly line up with the event. A higher-order time interpolation algorithm is used to solve for these positions.

Shipping Dimensions:

Vantage system= 20.41 kg (45lbs), 1.04 m (41 in) x 0.20 m (8 in) x 0.20 m (8 in)

Vantage System Case (sold separately) = 16.7 kg (37 lbs), 1.02 m (40 in) x 0.48 m (19 in) x 0.18 m (7 in)
